# Associations among environmental unpredictability, changes in resting-state functional connectivity, and adolescent psychopathology in the ABCD study

**DOI:** 10.1017/S0033291724001855

**Published:** 2024-11

**Authors:** Yumeng Yang, Tianjiao Kong, Feng Ji, Ran Liu, Liang Luo

**Affiliations:** 1 State Key Laboratory of Cognitive Neuroscience and Learning, Beijing Normal University, Beijing, China; 2 Faculty of Psychology, Institute of Developmental Psychology, Beijing Normal University, Beijing, China; 3 Department of Applied Psychology and Human Development, University of Toronto, Toronto, Canada

**Keywords:** ABCD study, cingulo-opercular network, default mode network, fronto-parietal network, psychopathology, unpredictability

## Abstract

**Background:**

Unpredictability is a core but understudied dimension of adversities and has been receiving increasing attention recently. The effects of unpredictability on psychopathology and the underlying neural mechanisms, however, remain unclear. It is also unknown how unpredictability interacts with other dimensions of adversities in predicting brain development and psychopathology of youth.

**Methods:**

We applied cluster robust standard errors to examine how unpredictability was associated with the developmental changes in resting-state functional connectivity (rsFC) of large-scale brain networks implicated in psychopathology, as well as the moderating role of deprivation, using data from the Adolescent Brain Cognitive Development (ABCD) study, which included four measurements from baseline (mean ± s.d. age, 119.13 ± 7.51 months; 2815 females) to 3-year follow-up (*N* = 5885).

**Results:**

After controlling for threat, unpredictability was associated with a smaller increase in rsFC within default mode network (DMN) and a smaller decrease in rsFC between cingulo-opercular network (CON) and DMN. Neighborhood educational deprivation moderated the associations between unpredictability and changes in rsFC within DMN and fronto-parietal network (FPN), as well as between CON and DMN. A smaller decrease in rsFC between CON and DMN mediated the association between unpredictability and externalizing problems. Neighborhood educational deprivation moderated the indirect pathway from unpredictability to externalizing problems via a smaller decrease in CON-DMN rsFC.

**Conclusions:**

Our findings shed light on the neural mechanisms underlying the associations between unpredictability and adolescents' psychopathology and the moderating role of deprivation, highlighting the significance of providing stable environment and abundant educational opportunities to facilitate optimal development.

Early-life adversities are highly prevalent and are associated with a wide range of negative consequences in behavioral and neural development of adolescents (Holland et al., 2020; North, Fox, & Doom, 2023; Rakesh et al., 2021a; Ramos et al., 2022). To better understand the specific mechanisms linking disparate adversities and development, accounting for the notion that different types of adversities often co-occur and may share common features, recent studies have increasingly adopted dimensional models, which generally include three dimensions: threat, deprivation, and unpredictability (Ellis, Figueredo, Brumbach, & Schlomer, 2009; McLaughlin, Sheridan, & Lambert, 2014; Sheridan & McLaughlin, 2014). Although all three dimensions are closely linked to children's negative developmental outcomes, the unique effects of unpredictability and its underlying mechanisms are still unclear compared with the other dimensions (Liu & Fisher, 2022; Wade, Wright, & Finegold, 2022), highlighting the need for further empirical investigation.

Unpredictability refers to spatial-temporal variation in threat or deprivation (Ellis, Sheridan, Belsky, & McLaughlin, 2022). Two perspectives, the ancestral cue and statistical learning perspectives, offer insights into the approaches of measuring unpredictability (Young, Frankenhuis, & Ellis, 2020). The ancestral cue perspective proposes that humans evolved to detect unpredictability; therefore, unpredictability can be assessed with cues (e.g. parental transition) that reliably indicate high unpredictability. Statistical learning perspective suggests that humans evaluate the level of unpredictability by integrating variations in lived experiences throughout development; therefore, unpredictability can be measured by collecting series data indicating statistical properties, such as variance and autocorrelation (Young et al., 2020). Moreover, based on topological approach and related studies, how children understand and interpret stressful experiences might shape their biological and psychosocial development, beyond the impact of exposure to stressful events (Baldwin & Degli Esposti, 2021; Smith & Pollak, 2021, 2022; Ugarte & Hastings, 2022). In this study, we adopted an ancestral cue perspective, focusing on children's perception of unpredictability, to examine the associations between unpredictability and subsequent neural and behavioral outcomes in adolescence.

According to the life history theory (Roff, 2002; Stearns, 1992), individuals tend to adopt faster life history strategies (e.g. early pubertal maturation, sexual behavior, and reproductive timing, as well as increased impulsivity and risk taking) to enhance evolutionary fitness in unpredictable environment (Belsky, Schlomer, & Ellis, 2012; Ellis et al., 2009). Although being evolutionarily adaptive, faster life history strategies are associated with adverse developmental outcomes in the long-term, especially incurring more risky and aggressive behaviors, which are typical types of externalizing problems (Ellis et al., 2009, 2022; Ellis, Shakiba, Adkins, & Lester, 2021; Martinez et al., 2022). The associations between unpredictability and internalizing problems, however, are less consistent (Farkas, Baptista, Speranza, Wyart, & Jacquet, 2024; Li & Belsky, 2022; Li, Sturge-Apple, Jones-Gordils, & Davies, 2022; Lindert et al., 2022; Spadoni et al., 2022). Informed by the life history theory, recent studies started to highlight the unique role of unpredictability in children's development above and beyond the other dimensions (Li et al., 2023; Liu & Fisher, 2022; Wang, Cao, Zheng, Chen, & Zhu, 2023); however, it remains uncertain *how* unpredictability shapes socioemotional development. Elucidating the underlying neural mechanisms will improve our understanding of the deep reasons behind the associations between unpredictability and diverse types of behavioral problems.

Different dimensions of adversity may be interactively associated with neurodevelopment and psychopathology. For example, social deprivation exacerbated the effects of childhood violence exposure on the development of amygdala-orbitofrontal cortex white matter connections (Goetschius et al., 2020). To our best knowledge, however, limited studies have examined the interactive effects of unpredictability and other dimensions of adversity on psychopathology and the neural underpinnings, hindering our understanding of the conditions under which unpredictability may have an impact. In a recent review, Colich, Rosen, Williams, and McLaughlin (2020) proposed that whether unpredictability associated with deviated development depends upon various features of the environment including resource availability. Therefore, deprivation, involving limited or reduced social and cognitive inputs from the environment (McLaughlin et al., 2014; Sheridan & McLaughlin, 2014), may moderate the associations between unpredictability and neural as well as socioemotional development. Moreover, the associations between adversity and brain development often differ by sex (Rakesh et al., 2021a; Rakesh, Allen, & Whittle, 2023), although it is uncertain of sex differences in unpredictability–neurodevelopment associations. As such, this study examined the moderating effects of deprivation and sex on the associations between unpredictability and neurodevelopment. Considering environmental unpredictability mainly reflected variations in threat/deprivation at the level of family in this study, we particularly focused on deprivation in the neighborhood context, as neighborhood is also an important aspect of environment (Rakesh, Seguin, Zalesky, Cropley, & Whittle, 2021b).

Resting-state functional connectivity (rsFC) provides a powerful approach to examine the neurobiological pathways linking adversities and child development (Daliri & Behroozi, 2014). Previous work has mainly focused on rsFC of the frontolimbic circuitry (e.g. Brieant, Sisk, & Gee, 2021; Kaiser et al., 2018), though more widespread networks may be associated with adversities (e.g. Rakesh et al., 2021a), such as the higher-order brain networks, including the cingulo-opercular network (CON), default mode network (DMN), and fronto-parietal network (FPN). The CON, encompassing the dorsal anterior cingulate cortex and bilateral anterior insula, consistently activates during tasks involving error detection and ongoing task management (Menon & Uddin, 2010). The DMN, comprising mainly midline cortical regions, such as the anterior medial prefrontal cortex and posterior cingulate cortex, activates when individuals are not focused on external tasks but instead are engaged in self-reflection or introspection (Raichle, 2015). The FPN, which consists of dorsolateral prefrontal and posterior parietal cortices, functions as a control network orchestrating behavior towards specific goals (Marek & Dosenbach, 2018). Moreover, connectivity between task-positive networks (typically activated during tasks, e.g. CON and FPN) and task-negative networks (typically deactivated during tasks, e.g. DMN; Yu et al., 2019) also has important functions. For example, connectivity between the FPN-B (subnetwork of FPN), dorsal attention network, CON and lateral DMN was associated with switching and inhibiting behaviors (Beaty, Cortes, Zeitlen, Weinberger, & Green, 2021); greater negative connectivity between FPN and DMN was linked to less mind wandering during tasks necessitating external attention (Deck et al., 2023; Kelly, Uddin, Biswal, Castellanos, & Milham, 2008). Aberrant rsFC, both within and between CON, DMN, and FPN, were implicated in children's internalizing and externalizing problems (Chahal, Miller, Yuan, Buthmann, & Gotlib, 2022; Rakesh et al., 2021a, 2021b). As such, our study mapped unpredictability-related psychopathology onto the rsFC within and between large-scale networks (i.e. CON, DMN, and FPN) to offer a more comprehensive view of the brain mechanisms linking adversity and psychopathology.

Moreover, the brain networks undergo dynamic restructuring in late childhood and adolescence (Fair et al., 2007; Grayson & Fair, 2017; Lin et al., 2008); however, the existing work of adversity has mostly relied on cross-sectional designs (e.g. Rakesh et al., 2021b), which cannot reveal how adversity impacts brain maturation over development. Normative developmental changes include positive associations between age and rsFC within networks (Truelove-Hill et al., 2020; but see Chahal et al., 2022), as well as negative associations between age and between-network connectivity (Stevens, 2016; but see Sanders et al., 2023). Deviations from the typical development of brain networks could be detected under pathological conditions (Dennis & Thompson, 2013; Rakesh et al., 2021a). The stress acceleration theory suggests that early-life adversity accelerates neural development, marked by faster maturation of the cortico-limbic circuits (Callaghan & Tottenham, 2016; Gee et al., 2013a, 2013b). Despite the widespread influences of adversity on brain, limited longitudinal work has examined the effects of adversity on changes in higher-order brain networks rsFC with results indicating both accelerating (Rakesh et al., 2023) and delaying effect (Rakesh et al., 2021a). The inconsistency highlights the need for additional longitudinal work, especially those with larger sample sizes (Chahal et al., 2022; e.g. Adolescent Brain Cognitive Development Study [ABCD]), to better elucidate how adversity, especially unpredictability, shapes the development of large-scale brain networks implicated in psychopathology.

In summary, this study had four major aims. First, we examined the effects of environmental unpredictability on the changes in rsFC within and between three major higher-order networks – CON, DMN, and FPN from baseline to 2-year follow-up. We hypothesized that rsFC within networks would increase and rsFC between networks would decrease from baseline to 2-year follow-up, with greater unpredictability accelerating these changes based on the stress acceleration theory; however, given prior limited and inconsistent findings, this hypothesis was exploratory. Second, we examined whether neighborhood deprivation moderated the associations between unpredictability and changes in rsFC of brain networks. We hypothesized that unpredictability would be associated with atypical development of network rsFC only when adolescents were also exposed to increased deprivation. As exploratory analyses, we also examined whether sex played a moderated role, as well as the three-way interaction of unpredictability, deprivation, and sex; we did not make specific hypotheses regarding sex differences and three-way interaction due to limited prior research. Third, we examined the mediating effects of changes in rsFC and hypothesized that unpredictability would be associated with atypical development of brain network rsFC, which in turn would increase adolescents' behavioral problems. Fourth, as exploratory analyses, we examined whether neighborhood deprivation moderated the indirect pathways from environmental unpredictability to adolescent behavioral problems through changes in rsFC.

## Methods

### Participants

Participants were from the ABCD study (https://abcdstudy.org/). The ABCD study is an ongoing longitudinal study that has recruited 11 868 children (9–10 years of age) from 21 study sites across the United States (Casey et al., 2018). We used baseline, 1-year, 2-year, and 3-year follow-up data from the 5.0 release. After excluding participants who did not have resting-state functional magnetic resonance imaging (rsfMRI) data, or whose rsfMRI data were recommended for exclusion by the ABCD analytic core at baseline and 2-year follow-up, the final sample consisted of *N* = 5885 participants (see Table 1 for demographic information). All participants provided informed consent or assent, detailed ethics information can be found in Clark et al. (2018).

**Table 1.** Demographic information

Characteristic		*n* or *M* ± s.d.
*N* total (*n* female)		5885 (2815)
Age at baseline, months		119.13 ± 7.51
Scanner type at 2-year follow-up	SIEMENS	3858
	Philips	1464
	GE	563
Framewise displacement of rsfMRI at 2-year follow-up, mm		0.16 ± 0.15
Race	Asian	101
	Black	678
	Hispanic	1138
	White	3359
	Other	609
Environmental unpredictability at 1-year follow-up		2.95 ± 3.53
Neighborhood deprivation at baseline	Education	0.03 ± 0.07
	Health and environment	0.03 ± 0.04
	Social and economic	0.07 ± 0.19
	Overall	0.01 ± 0.03
Internalizing problems at 3-year follow-up		5.20 ± 5.92
Externalizing problems at 3-year follow-up		3.88 ± 5.36

### Environmental and behavioral data

#### Environmental unpredictability and threat

We used a subset of items from the Life Events Scale (LES; Grant, Compas, Thurm, McMahon, & Gipson, 2004; Hoffman et al., 2019; Tiet et al., 1998) to measure environmental unpredictability at 1-year follow-up. LES is a 33-item questionnaire measuring children's stressful life events. We operationalized and measured unpredictability from ancestral cue perspective (Young et al., 2020) by using classic items similar to those employed in previous research (see online Supplementary information for all selected 16 items; Belsky et al., 2012; Mittal, Griskevicius, Simpson, Sung, & Young, 2015). If children reported having experienced the stressful event and perceiving it as bad, the score of the degree of disruption was used, where 0 = not at all, 1 = a little, 2 = some, 3 = a lot, otherwise the score was indicated as 0. Then we computed the sum score of the degree of disruption. Therefore, the higher the total score, the greater level of environmental unpredictability the child perceived.

Additionally, we selected four items pertaining to the experiences or witness of severe trauma events to measure perceived environmental threat, consistent with measures employed in previous research (Dennison et al., 2019; see online Supplement information for selected items). The higher the total score, the greater level of environmental threat the child perceived.

#### Neighborhood deprivation

We used the Child Opportunity Index (COI) 2.0 to measure neighborhood deprivation (Noelke et al., 2020). The COI 2.0 was derived from residential geocodes based on primary address information provided at baseline (Acevedo-Garcia et al., 2014). For detailed information on the computational procedure and ethical considerations, please refer to the online Supplementary information and Fan et al. (2021). The index scores captured neighborhood resources and conditions from three domains: education; health and environment; social and economic. Using the composite score may hide the distinct effect of different types of deprivation, which were revealed in previous studies (Dennison et al., 2019). Therefore, we used the weighted average *z*-scores of each domain and the overall COI (weighted average of three domain averaged *z*-scores), nationally normed. The lower the *z*-scores, the greater levels of deprivation adolescents experienced.

#### Internalizing and externalizing problems

We used the Child Behavior Checklist (CBCL; Achenbach, 2018; Karcher & Barch, 2021), which measured children's psychopathology and behaviors over the past 6 months. Parents reported at baseline and at 3-year follow-up. Given we examined sex differences and longitudinal changes in this study, we used raw scores rather than sex- and age-corrected T-scores, from the internalizing and externalizing symptom subscales, which were rated on a 3-point Likert-type scale ranging from ‘0 = not true’ to ‘2 = very true’.

### Imaging data

**Figure 1.**
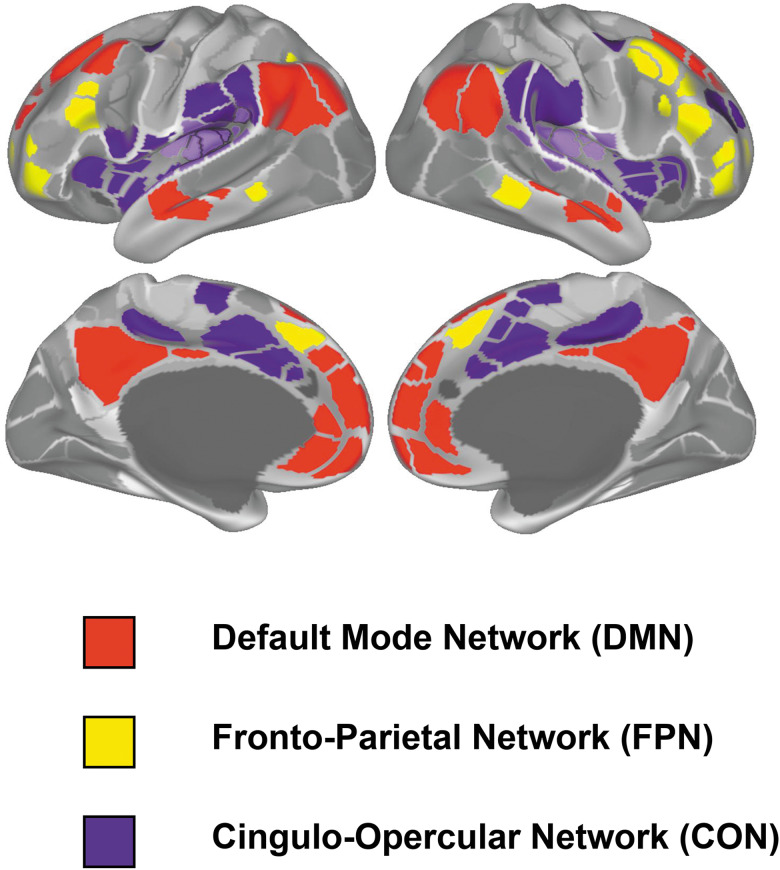
Three higher-order brain networks in Gordon cortical network parcellation.

Participants underwent neuroimaging scans using standardized protocols across sites (see Casey et al. [2018] for detailed imaging procedures). In this study, we used rsfMRI data at baseline and at 2-year follow-up. The pre-processed time courses for each participant were mapped onto the cortical surface, using the standardized ABCD pipeline (Hagler et al., 2019). Using these time courses, within- and between-network connectivity (Pearson correlation) were computed based on the Gordon functional parcellation (Gordon et al., 2016), and then Fischer *z* transformed. We focused on the changes in rsFC within and between the three major networks: CON, DMN, and FPN (Fig. 1).

### Statistical methods

First, to test the associations between environmental predictability and rsFC, we conducted models using cluster robust standard errors (CR-SEs; the highest level – site ID was modeled as clustering variable, TYPE = COMPLEX; McNeish, Stapleton, & Silverman, 2017) to account for the clustering structure (i.e. multiple children from the same family and site) in Mplus Version 7.4 (Muthén & Muthén, 1998–2014), with network rsFC values as outcomes (in separate models) and environmental unpredictability as the predictor. Second, we examined the moderating roles of neighborhood deprivation and sex on the associations between environmental unpredictability and rsFC. If the moderating effects were significant, Johnson–Neyman method was used to assist further interpretation of moderating effects (Johnson & Fay, 1950). Furthermore, we examined the mediating roles of rsFC linking environmental unpredictability and adolescents' behavioral problems, using 95% bias-corrected confidence intervals (CIs) with 5000 bootstrap samples in the mediation analysis. We also examined the potential moderating effect of neighborhood deprivation on the indirect paths from environmental unpredictability to behavioral problems via rsFC.

We controlled for threat, sex, race, age, scanner type, and mean framewise displacement when predicting rsFC at 2-year follow-up. We also controlled for rsFC at baseline; therefore, the outcome was residualized to eliminate baseline connectivity effects, leaving only variances attributable to developmental change. In models predicting behavioral problems at 3-year follow-up, we also covaried threat, sex, race, age, and internalizing/externalizing problems at baseline. In all models, full information maximum likelihood estimation was utilized to address missing data in the study variables (please see online Supplementary Table S1 for missing variables, rates, and patterns). We controlled for multiple comparisons using the false discovery rate (*p* < 0.05).

## Results

### Correlations between study variables and changes in rsFC

Correlations between all study variables were shown in online Supplementary Fig. S1 (see online Supplementary information). Pair-sample *t* test indicated that rsFC within networks increased and rsFC between networks decreased from baseline to 2-year follow-up (online Supplementary Table S2).

### Associations between environmental unpredictability and changes in rsFC

We found that greater environmental unpredictability was associated with a smaller increase in rsFC within DMN and a smaller decrease in rsFC between CON and DMN (*B* = −0.042, s.e. = 0.010, *p* < 0.001, 95% CI [−0.064 to −0.025]; *B* = 0.035, s.e. = 0.008, *p* < 0.001, 95% CI [0.021–0.052]; online Supplementary Table S3).

### Moderating roles of neighborhood deprivation and sex

We found that neighborhood educational deprivation moderated the associations between environmental unpredictability and rsFC within DMN, FPN, as well as between CON and DMN (*B* = 0.034, s.e. = 0.012, *p* = 0.004, 95% CI [0.011–0.057]; *B* = 0.025, s.e. = 0.008, *p* = 0.003, 95% CI [0.009–0.041]; *B* = −0.028, s.e. = 0.010, *p* = 0.004, 95% CI [−0.048 to −0.009]; online Supplementary Table S4).

**Figure 2.**
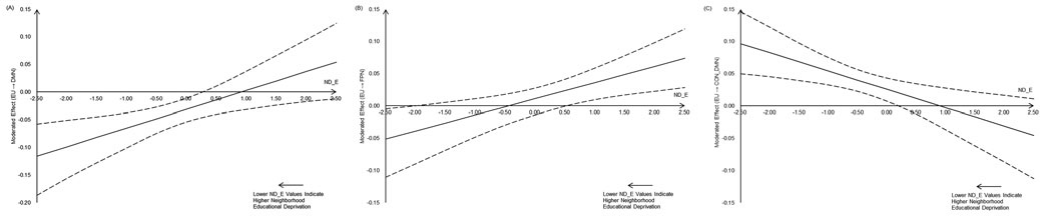
The moderating effect of neighborhood educational deprivation (ND_E; lower ND_E values indicate higher neighborhood educational deprivation) on the associations between environmental unpredictability (EU) and changes in rsFC within DMN (a), FPN (b), as well as between CON and DMN (c). CON, cingulo-opercular network; DMN, default mode network; FPN, fronto-parietal network.

Johnson–Neyman plots (Fig. 2) showed that when the level of neighborhood educational deprivation was high, greater environmental unpredictability was associated with a smaller increase in rsFC within DMN and a smaller decrease in rsFC between CON and DMN; when the level of neighborhood educational deprivation was low, however, the association was not significant. We also found that when the level of neighborhood educational deprivation was extremely high, greater environmental unpredictability was associated with a smaller increase in rsFC within FPN; when the level of neighborhood educational deprivation was moderate, the association was not significant; when the level of neighborhood educational deprivation was low, greater environmental unpredictability was associated with a greater increase in rsFC within FPN.

The moderating effects of other domains of deprivation, overall deprivation, sex, or the three-way interactions among environmental unpredictability, neighborhood deprivation, and sex were not significant (online Supplementary Tables S5–S12).

### Mediating roles of changes in rsFC between environmental unpredictability and adolescents' behavioral problems

**Figure 3.**
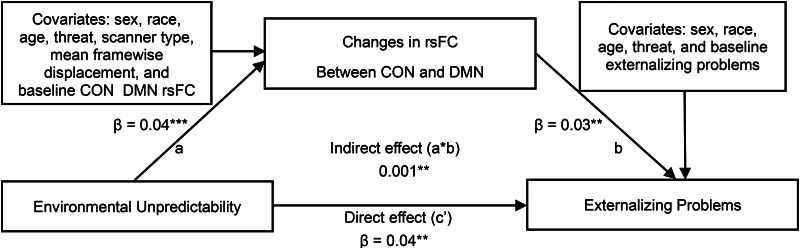
The mediating effect of changes in CON-DMN rsFC between environmental unpredictability and adolescents' externalizing problems. CON, cingulo-opercular network; DMN, default mode network; FPN, fronto-parietal network. The parameter estimates in the table are all standardized. *p < 0.05, **p < 0.01, ***p < 0.001.

Based on the significant results of the associations between environmental unpredictability and changes in rsFC, we conducted further analyses to test if rsFC within DMN and between CON and DMN mediated the associations between environmental unpredictability and internalizing as well as externalizing problems, respectively. The results showed that greater environmental unpredictability was associated with a smaller decrease in rsFC between CON and DMN, which predicted more externalizing problems (indirect effect = 0.001, s.e. = 0.000, *p* = 0.007, 95% CI [0.001–0.002]; Fig. 3 and online Supplementary Table S13).

### Moderating role of neighborhood educational deprivation in the mediation model

**Figure 4.**
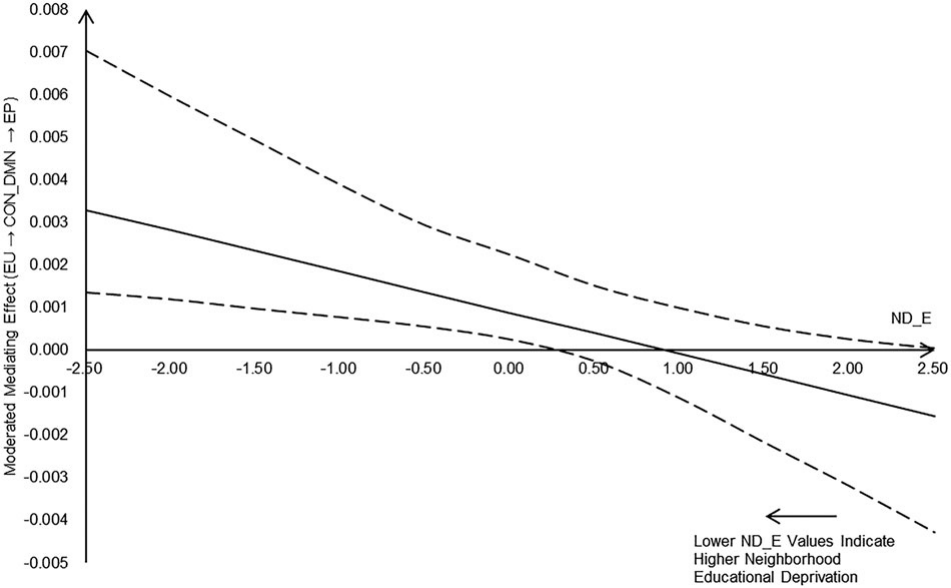
The moderating effect of neighborhood educational deprivation (ND_E; lower ND_E values indicate higher neighborhood educational deprivation) on the indirect path from environmental unpredictability (EU) to externalizing problems (EP) via changes in CON-DMN rsFC. CON, cingulo-opercular network; DMN, default mode network; FPN, fronto-parietal network.

Considering the moderating effect of neighborhood educational deprivation on the associations between environmental unpredictability and rsFC within DMN, FPN, as well as between CON and DMN, we conducted moderated mediation models to examine whether neighborhood educational deprivation moderated the paths from unpredictability to adolescents' behavioral problems through rsFC of these networks. Results showed that neighborhood educational deprivation moderated the indirect path from environmental unpredictability to internalizing/externalizing problems via rsFC between CON and DMN (moderated mediating effect = −0.001, s.e. = 0.001, *p* = 0.028, 95% CI [−0.003 to −0.001]; moderated mediating effect = −0.002, s.e. = 0.001, *p* = 0.023, 95% CI [−0.004 to −0.001]; Fig. 4 and online Supplementary Table S14).

Particularly, only when the level of neighborhood educational deprivation was high, greater environmental unpredictability was associated with a smaller decrease in rsFC between CON and DMN, which was linked to greater externalizing problems and internalizing problems; however, the result pertaining to internalizing problems became non-significant after handling outliers (see sensitivity analysis), thus, we did not provide further explanations for this result.

### Sensitivity analysis

To assess the potential bias introduced by outliers in model estimates, we conducted a sensitivity analysis using the winsorization method. Specifically, observations with values exceeding 4 standard deviations from the mean on any model variables were replaced with the nearest maximum or minimum value (Dixon & Yuen, 1974; Hair, Black, Babin, & Anderson, 2013). The results were highly consistent with those obtained prior to outlier treatment, however, one moderated mediation model (i.e. environmental unpredictability and neighborhood deprivation interactively predicted internalizing problems via changes in rsFC between CON and DMN) became non-significant after handling outliers (moderated mediating effect = −0.001, s.e. = 0.001, *p* = 0.030, 95% CI [−0.003 to −0.000]; see online Supplementary Tables S15–S18).

## Discussion

This is the first study to examine the associations between environmental unpredictability and longitudinal changes in rsFC of three higher-order networks (i.e. CON, DMN, FPN) implicated in internalizing and externalizing problems of adolescents, as well as the moderating effects of neighborhood deprivation and sex.

First, typical developmental patterns of networks in this study included increased rsFC within networks (i.e. CON, DMN, and FPN) and decreased rsFC between networks (i.e. CON and DMN, CON and FPN, DMN and FPN), which were in line with existing work (Stevens, 2016; Truelove-Hill et al., 2020). After controlling for threat, environmental unpredictability was negatively associated with changes in rsFC within DMN, and positively associated with changes in rsFC between CON and DMN, suggesting that unpredictability might be associated with delayed development of higher-order brain networks, which was consistent with previous research (Philip et al., 2013; Rakesh et al., 2021a). However, this finding was inconsistent with the stress acceleration hypothesis, which might be attributable to the diverse functions and developmental trajectories of these different neural circuits. Frontolimbic circuits mainly function in emotional processing and regulation (Callaghan & Tottenham, 2016), while higher-order brain networks are primarily involved in cognitive control (especially CON and FPN; Marek & Dosenbach, 2018; Menon & Uddin, 2010). Moreover, higher-order brain networks (i.e. CON, DMN, and FPN) reorganize in late childhood and into adulthood (Fair et al., 2007; Grayson & Fair, 2017; Lin et al., 2008), while limbic regions (e.g. hippocampus and amygdala) develop earlier in life (i.e. early childhood and adolescence; Curlik, DiFeo, & Shors, 2014; DiFeo & Shors, 2017; Scherf, Smyth, & Delgado, 2013; Tottenham & Galván, 2016); therefore, unpredictability may exert complex and varied impacts on the development of these different neural circuits.

Importantly, a smaller decrease in CON-DMN rsFC mediated the associations between environmental unpredictability and adolescent externalizing problems. Children with disrupted segregation between CON and DMN showed less intertemporal decision-making (Chen, Guo, Suo, & Feng, 2018), indicating that they might take more risky behaviors disregarding for long-term development. The impairment of connectivity between CON and DMN, reflecting unbalanced patterns between task-positive networks (e.g. CON) and task-negative networks (e.g. DMN), which were associated with dysfunction of executive function, emotional dysregulation, and more externalizing problems (Sato et al., 2015; Yu et al., 2019). The findings might partly explain why greater unpredictability was associated with increased externalizing problems but not internalizing problems, as the life history theory indicating that unpredictability was generally linked to faster life history strategies, including increased risky sexual and aggressive behaviors (Ellis et al., 2021), higher delay discounting, more impulsivity, and externalizing behaviors (Martinez et al., 2022). This study contributes to the field by shedding light on the neural mechanisms underlying the effect of unpredictability on faster life-history strategies.

Furthermore, consistent with our hypothesis, the co-occurrence of unpredictability and deprivation predicted the lowest rsFC within DMN and FPN, as well as the highest rsFC between CON and DMN, indicating that deprivation exacerbated the adverse effect of unpredictability on adolescents' neurodevelopment, which was consistent with prior research (Goetschius et al., 2020). We extended previous research by first showing the interactive effect of environmental unpredictability and neighborhood deprivation on the developmental patterns of brain network rsFC in adolescents. More importantly, we further revealed that a smaller decrease in CON-DMN rsFC was linked to increased externalizing problems one year later. The moderated mediation model provided a comprehensive picture demonstrating how interaction of unpredictability and deprivation was associated with adolescents' developmental outcomes and the potential neural mechanisms.

Interestingly, we also found that when there were abundant neighborhood educational resources, environmental unpredictability was positively associated with increased rsFC within FPN. The FPN initiates and regulates behavior in a purposeful and goal-oriented manner (Marek & Dosenbach, 2018), thus may play a significant role in the process of prioritization and coordination of life history events (short-term survival or long-term development). When numerous educational resources were available in the neighborhood, environmental unpredictability might be associated with an accelerated development of FPN, facilitating the utilization of opportunities or resources for survival and growth, avoiding loss when waiting for long-term rewards in unpredictable environment (Mell, Baumard, & André, 2021). Interestingly, the changes within FPN were not significantly associated with behavioral problems. The organization within FPN follows different developmental trajectories (connectivity increases within prefrontal regions while decreases within parietal regions; Hwang, Velanova, & Luna, 2010; Marek & Dosenbach, 2018); thus, certain factors (e.g. regions) may moderate the association between changes in FPN rsFC and behavioral problems. Actually, although not significant, we found that increased rsFC within FPN was associated with less internalizing and externalizing problems in trend. As such, unpredictability and simultaneous abundant neighborhood educational opportunities were associated with an accelerated development of FPN, which might have evolutionary significance by reducing internalizing and externalizing problems in the short term, although the long-term effects were still unclear.

Interestingly, only neighborhood deprivation in education domain moderated the associations between environmental unpredictability and rsFC within DMN, FPN, as well as between CON and DMN, as opposed to the other domains of neighborhood deprivation, reflecting the unique role of educational aspect of neighborhood deprivation. Indicators of neighborhood educational deprivation (e.g. early care and education settings) had profound and lasting effects on individuals' educational attainment and many other developmental outcomes (Bozick & DeLuca, 2011; Leventhal & Brooks-Gunn, 2000; Magnuson & Duncan, 2016). Lower educational opportunities, or limited cognitive inputs in family were associated with poor cue attention, working memory performance, and blunting activation of the parietal and prefrontal cortex among children and adolescents (Rosen, Meltzoff, Sheridan, & McLaughlin, 2019; Sheridan, Peverill, Finn, & McLaughlin, 2017). Our findings extended the literature by demonstrating the adverse effects of educational and cognitive deprivation in neighborhood environment.

We did not find sex difference in the associations between unpredictability and rsFC of brain networks, which was inconsistent with prior research indicating male-specific associations between maltreatment and neglect as well as network rsFC alterations (Rakesh et al., 2021a, 2023). As such, the sex differences in associations between adversity and neurodevelopment might be different for unpredictability and other dimensions of adversity. Limited studies have examined sex differences in rsFC that is associated with unpredictability; therefore, more related research is needed in the future.

This study had many theoretical and methodological strengths, including employing a longitudinal design and a multi-level approach to reveal how household unpredictability interacted with neighborhood deprivation in predicting individual's developmental changes in rsFC and psychopathology, using a large and nationally representative sample to attain more reproducible and generalizable results (Maleki, Ovens, McQuillan, & Kusalik, 2019), and applying advanced statistical methods to conduct analyses and handle missing values. Some limitations, however, should also be mentioned. First, we only tested how environmental unpredictability was associated with neurodevelopment and behavioral outcomes over a 3-year period. With the continuation of ABCD study, we hope to examine the prolonged effects of unpredictability as the participants enter middle and late adolescence. Second, we measured unpredictability from an ancestral cue perspective, which made it difficult to distinguish between threat/deprivation and unpredictability (Ellis et al., 2022), although we controlled for threat in all models. Future research can combine the perspectives of statistical learning and ancestral cue (Young et al., 2020). Third, COI 2.0 estimated neighborhood resources and conditions from 2010 to 2015, but residential addresses were obtained at baseline from 2016 to 2018; therefore, COI 2.0 might not accurately reflect the current neighborhood environments if participants moved or the neighborhood condition had changed significantly, which might confound the findings of this study, warranting cautious in interpreting the moderating effects of neighborhood deprivation. Future studies that use residential address-linked metrics may consider employing quality control or utilizing updated COI 3.0 to further validate our findings.

In sum, this study improved our understanding of the normative developmental patterns of rsFC within and between CON, DMN, and FPN. More importantly, the study shed light on the effects of unpredictability on changes in rsFC of higher-order brain networks implicated in psychopathology and the moderating role of deprivation. It is crucial for family, neighborhood, and society to provide stable and predictable environments as well as abundant educational opportunities to promote the normative neurodevelopment of children and adolescents, thereby preventing the occurrence of externalizing problems.

## Supporting information

Yang et al. supplementary material Yang et al. supplementary material

## Supplementary material

The supplementary material for this article can be found at https://doi.org/10.1017/S0033291724001855
