# Supplementary material for: Associations among environmental unpredictability, changes in resting-state functional connectivity, and adolescent psychopathology in the ABCD study
Source: Psychol Med. 2024 Nov 18;54(15):4119–28. doi: 10.1017/S0033291724001855 (PMC11650185; doi:10.1017/S0033291724001855)
Supplement: Yang et al. supplementary material [file S0033291724001855sup001.docx]

**Associations Among Environmental Unpredictability, Changes in Resting-State Functional Connectivity, and Adolescent Psychopathology in the ABCD study**

***Supplementary Information***

**We operationalized and measured unpredictability from ancestral cue perspective (Young et al., 2020) by using classic items similar to those employed in previous research. For example, Belsky et al. (2012) assessed unpredictability using three measures, containing paternal transitions, household moves, and parental employment transitions; Mittal, Griskevicius, Simpson, Sung, and Young (2015) measured unpredictability using three items from the Life Stress Inventory (Cochrane & Robertson, 1973): changes in employment status during the prior year (e.g., periods of unemployment), changes in residence during the prior year (e.g., moving to a different house/apartment) and people moving in and out of the house during the prior year (e.g., mother’s romantic partners moving in or out; an immediate family member receiving a jail sentence). Referring to these measures, we selected 16 items from the Life Events Scale assessing environmental unpredictability from three sources: family (13 items, e.g., “negative change in parent's financial situation”), friend (2 items, e.g., “lost a close friend”) and school (1 item, i.e., “attended a new school”). The selected items for measuring environmental unpredictability in Life Events Scale are as follows:**

1. Someone in family died?

2. Lost a close friend?

3. Negative change in parent's financial situation?

4. Parents argued more than previously?

5. Mother/father figure lost job?

6. One parent was away from home more often?

7. Someone in the family was arrested?

8. Close friend died?

9. Brother or sister left home?

10. Parents separated or divorced?

11. Attended a new school?

12. Family moved?

13. One of the parents/caregivers went to jail?

14. Got new stepmother or stepfather?

15. Parent/caregiver got a new job?

16. Got new brother or sister?

**Selected Items for Measuring Environmental Threat in Life Events Scale:**

1. Saw crime or accident?
2. You got seriously sick?
3. You got seriously injured?
4. Was a victim of crime/violence/assault?

Children indicated whether they had experienced each stressor, if so, they were asked three sub-questions:

(1) Did this happen in the past year?

(2) Was this a good or bad experience?

(3) How much did the event affect you?

We calculate the total score for the third question (degree of disruption) of the corresponding items to measure environmental unpredictability and threat children percieved.

**Brief Introduction to Technical Documentation of COI 2.0 (Fan et al., 2021;** **Noelke et al., 2020)**

The Child Opportunity Index (COI) 2.0 assesses the resources and conditions within neighborhoods crucial for children's healthy development. This index covers virtually all neighborhoods in the 50 U.S. states and Washington, D.C., offering data for two time points: 2010 and 2015.

All component indicators were assessed at the census tract level using consistent 2010 census tract definitions for both COI 2.0 time frames (2010 and 2015). Census tracts align with the Census Bureau's definition of neighborhoods. The specific year or range of years covered by each indicator varies. Noelke et al. (2020) aimed to obtain either single-year data for 2010 and 2015 or multi-year averages centered around those years.

With 29 indicators spanning three domains—education, health and environment, and social and economic factors—COI 2.0 provides a thorough assessment. Specifically, indicators in education domain contain early childhood education, elementary education, secondary and postsecondary education, educational and social resources; indicators in health and environment domain contain healthy environments, toxic exposures, and health resources; indicators in social and economic domain contain economic opportunities, economic and social resources, with abundant scientific rationale for including them.

The COI incorporates indicators measured using various scales, such as counts, percentages, or U.S. dollars. To create an index combining indicators measured on different scales, we standardized the raw values of each indicator. Next, to aggregate different indicators within the same domain, the team assigned weights to each indicator. Indicator weights were calculated and validated using data from three sources, encompassing census tract-level metrics of adult health and economic outcomes. These sources include indicators of intergenerational social mobility sourced from the Opportunity Atlas, health metrics from the RWJF 500 Cities Project, and life expectancy statistics from the Centers for Disease Control and Prevention (CDC). The team calculated the average association between each component indicator of the COI and four outcome indicators. These average associations were then adjusted by a domain-specific constant, enhancing the weight of weakly associated indicators and reducing the weight of strongly associated ones. After determining the weights, the team multiplied each standardized indicator by its respective weight and summed them to compute domain scores for both time periods. This process was repeated to calculate the overall COI scores, where the outcomes were regressed on the domain scores to calculate the weights and overall COI score.

Primary residential addresses were obtained in person from the caregiver at baseline, conducted from October 2016 to October 2018. In cases where a child spent less than 80% of their time at the primary address, the Research Assistant recorded up to two additional current addresses. Subsequently, ABCD linked these addresses to corresponding neighborhoods (census tract) to attain the COI 2.0.

Table S1. Missing Data Variables, Rates, and Patterns.

| Variables | Missing Counts | Missing Rates (%) | Little’s MCAR |
| --- | --- | --- | --- |
| Environmental Unpredictability | 105 | 1.8 | χ^2^(73)=178.885  *p* < 0.05 |
| Neighborhood Educational Deprivation | 458 | 7.8 |  |
| Neighborhood Health Deprivation | 458 | 7.8 |  |
| Neighborhood Socioeconomic Deprivation | 458 | 7.8 |  |
| Overall Neighborhood Deprivation | 458 | 7.8 |  |
| CON | 8 | 0.1 |  |
| DMN | 8 | 0.1 |  |
| FPN | 8 | 0.1 |  |
| CON_DMN | 8 | 0.1 |  |
| CON_FPN | 8 | 0.1 |  |
| DMN_FPN | 8 | 0.1 |  |
| Internalizing Problems | 1087 | 18.5 |  |
| Externalizing Problems | 1087 | 18.5 |  |

Note. CON = average correlations within the Cingulo-Opercular Network, DMN = average correlations within the Default Mode Network, FPN = average correlations within the Fronto-Parietal Network, CON_DMN = average correlations between Cingulo-Opercular Network and Default Mode Network, CON_FPN = average correlations between Cingulo-Opercular Network and Fronto-Parietal Network, DMN_FPN = average correlations between Default Mode Network and Fronto-Parietal Network. Even if missingness is nonignorable, FIML is considered reasonable as long as the model includes correlates of missingness (Graham, 2003).


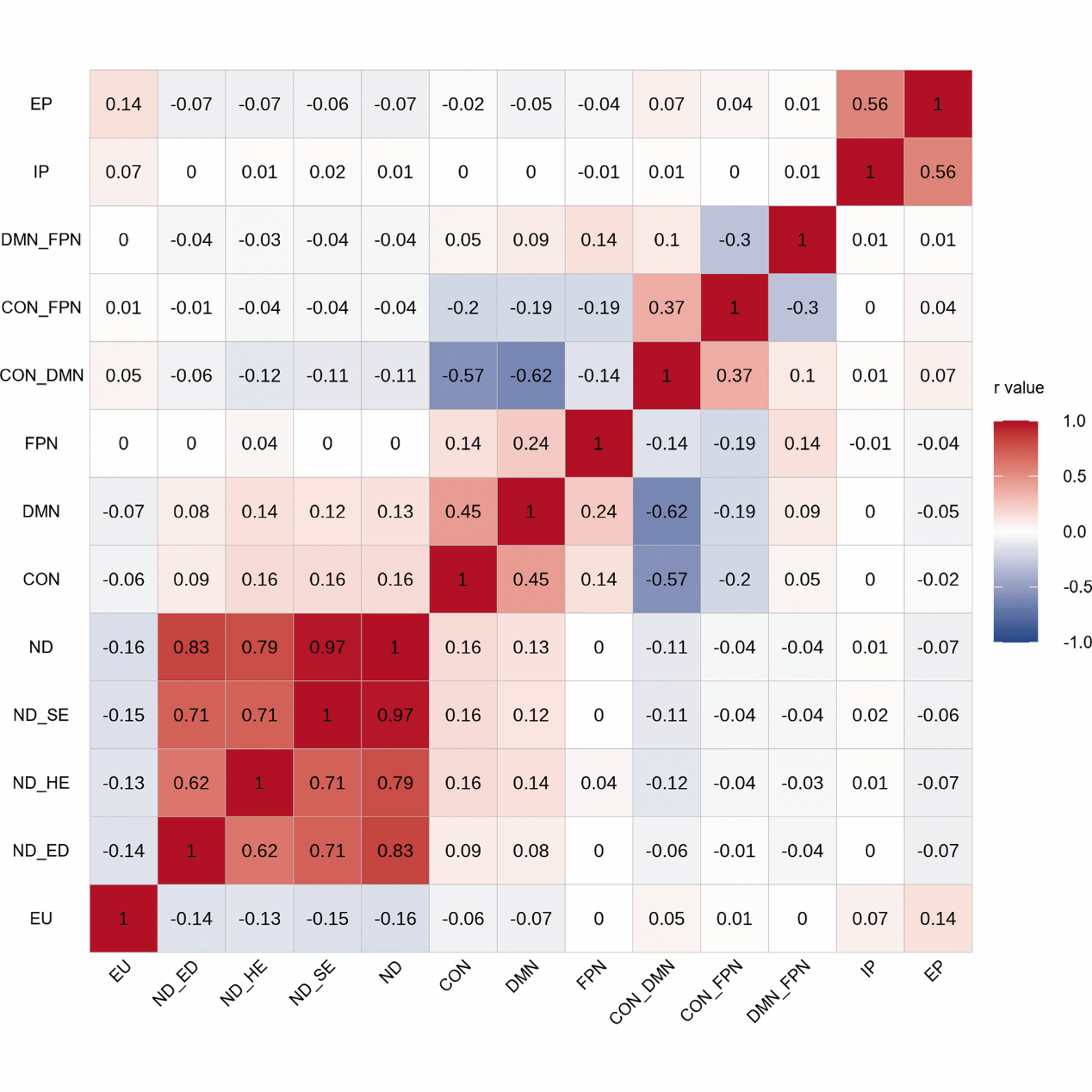


Figure S1. Correlation coefficients for study variables. EU = Environmental Unpredictability, ND_E = Neighborhood Deprivation in Education domain, ND_HE = Neighborhood Deprivation in Health and Environment domain, ND_SE = Neighborhood Deprivation in Social and Economic domain, ND = Overall Neighborhood Deprivation, CON = average correlations within the Cingulo-Opercular Network, DMN = average correlations within the Default Mode Network, FPN = average correlations within the Fronto-Parietal Network, CON_DMN = average correlations between Cingulo-Opercular Network and Default Mode Network, CON_FPN = average correlations between Cingulo-Opercular Network and Fronto-Parietal Network, DMN_FPN = average correlations between Default Mode Network and Fronto-Parietal Network, IP = Internalizing Problems, EP = Externalizing Problems.

Table S2. Pair-Sample *T*-test for Changes in rsFC of Brian Networks.

| Networks | *M*±*SD* (baseline) | *M*±*SD* (2-year follow-up) | *MD*±*SD* (0-2) | *95% CI of MD* | *t* | *df* | *Cohen’s d* | *p*  (FDR-corrected) |
| --- | --- | --- | --- | --- | --- | --- | --- | --- |
| CON0-CON2 | 0.31 ± 0.07 | 0.31 ± 0.07 | -0.00±0.07 | [-0.00, -0.00] | -2.94 | 5843 | 0.04 | 0.004 |
| DMN0-DMN2 | 0.24 ± 0.06 | 0.25 ± 0.06 | -0.01±0.06 | [-0.01, -0.01] | -10.52 | 5845 | 0.14 | < 0.001 |
| FPN0-FPN2 | 0.21 ± 0.06 | 0.22 ± 0.06 | -0.01±0.06 | [-0.01, -0.01] | -13.64 | 5845 | 0.18 | < 0.001 |
| CON_DMN0-CON_DMN2 | -0.11 ± 0.05 | -0.12 ± 0.06 | 0.01±0.06 | [0.01, 0.01] | 14.12 | 5843 | 0.18 | < 0.001 |
| CON_FPN0-CON_FPN2 | -0.01 ± 0.04 | -0.02 ± 0.05 | 0.00±0.05 | [0.00, 0.00] | 5.59 | 5843 | 0.07 | < 0.001 |
| DMN_FPN0-DMN_FPN2 | 0.05 ± 0.04 | 0.05 ± 0.04 | 0.00±0.05 | [0.00, 0.00] | 2.32 | 5845 | 0.03 | 0.020 |

Note. CON = average correlations within the Cingulo-Opercular Network, DMN = average correlations within the Default Mode Network, FPN = average correlations within the Fronto-Parietal Network, CON_DMN = average correlations between Cingulo-Opercular Network and Default Mode Network, CON_FPN = average correlations between Cingulo-Opercular Network and Fronto-Parietal Network, DMN_FPN = average correlations between Default Mode Network and Fronto-Parietal Network. And the number following the abbreviation of the variable name represents the time of data collection, 0 = baseline, 2 = 2-year follow-up.

Table S3. Associations between Environmental Unpredictability and Changes in rsFC.

| Independent Variable and Covariates | Dependent Variable | Unstandardized | | | | Standardized | | | | |  |
| --- | --- | --- | --- | --- | --- | --- | --- | --- | --- | --- | --- |
|  |  | Coefficient | *SE* | *95% CI* | *p* | | Coefficient | *SE* | *95% CI* | *p* | |
| Environmental Unpredictability | CON2 | 0.000 | 0.000 | [-0.001, 0.000] | 0.087 | | -0.021 | 0.012 | [-0.047, 0.001] | 0.084 | |
| Threat |  | -0.001 | 0.000 | [-0.002, 0.000] | 0.094 | | -0.019 | 0.011 | [-0.039, 0.002] | 0.084 | |
| Scanner Type |  | -0.002 | 0.002 | [-0.007, 0.002] | 0.354 | | -0.021 | 0.019 | [-0.057, 0.017] | 0.268 | |
| Framewise Displacement |  | -0.043 | 0.007 | [-0.058, -0.029] | < 0.001 | | -0.091 | 0.012 | [-0.113, -0.064] | < 0.001 | |
| Age2 |  | 0.000 | 0.000 | [0.000, 0.000] | 0.012 | | 0.024 | 0.009 | [0.004, 0.042] | 0.012 | |
| Race |  | -0.002 | 0.001 | [-0.003, -0.001] | 0.005 | | -0.033 | 0.011 | [-0.057, -0.012] | 0.004 | |
| Sex |  | 0.005 | 0.002 | [0.001, 0.009] | 0.010 | | 0.037 | 0.014 | [0.006, 0.063] | 0.010 | |
| CON0 |  | 0.507 | 0.011 | [0.485, 0.526] | < 0.001 | | 0.510 | 0.012 | [0.485, 0.531] | < 0.001 | |
| Environmental Unpredictability | DMN2 | -0.001 | 0.000 | [-0.001, 0.000] | < 0.001 | | -0.042 | 0.010 | [-0.064, -0.025] | < 0.001 | |
| Threat |  | 0.000 | 0.000 | [-0.001, 0.000] | 0.376 | | -0.010 | 0.011 | [-0.031, 0.012] | 0.375 | |
| Scanner Type |  | -0.009 | 0.006 | [-0.025, -0.002] | 0.187 | | -0.095 | 0.046 | [-0.209, -0.031] | 0.038 | |
| Framewise Displacement |  | -0.034 | 0.008 | [-0.050, -0.020] | < 0.001 | | -0.085 | 0.015 | [-0.113, -0.054] | < 0.001 | |
| Age2 |  | 0.000 | 0.000 | [0.000, 0.000] | 0.169 | | 0.016 | 0.012 | [-0.005, 0.040] | 0.167 | |
| Race |  | -0.002 | 0.001 | [-0.003, -0.001] | < 0.001 | | -0.041 | 0.012 | [-0.064, -0.020] | < 0.001 | |
| Sex |  | 0.009 | 0.001 | [0.007, 0.011] | < 0.001 | | 0.078 | 0.009 | [0.061, 0.096] | < 0.001 | |
| DMN0 |  | 0.519 | 0.030 | [0.452, 0.562] | < 0.001 | | 0.500 | 0.028 | [0.433, 0.538] | < 0.001 | |
| Environmental Unpredictability | FPN2 | 0.000 | 0.000 | [0.000, 0.000] | 0.446 | | 0.007 | 0.010 | [-0.016, 0.023] | 0.446 | |
| Threat |  | 0.000 | 0.000 | [-0.001, 0.000] | 0.222 | | -0.014 | 0.011 | [-0.037, 0.008] | 0.225 | |
| Scanner Type |  | -0.002 | 0.003 | [-0.008, 0.002] | 0.506 | | -0.021 | 0.024 | [-0.068, 0.025] | 0.383 | |
| Framewise Displacement |  | -0.030 | 0.0090 | [-0.045, -0.013] | 0.001 | | -0.077 | 0.017 | [-0.105, -0.039] | < 0.001 | |
| Age2 |  | 0.000 | 0.000 | [0.000, 0.000] | 0.305 | | 0.011 | 0.011 | [-0.012, 0.033] | 0.306 | |
| Race |  | -0.001 | 0.001 | [-0.002, 0.000] | 0.220 | | -0.015 | 0.012 | [-0.039, 0.008] | 0.226 | |
| Sex |  | 0.007 | 0.001 | [0.004, 0.010] | < 0.001 | | 0.060 | 0.013 | [0.035, 0.083] | < 0.001 | |
| FPN0 |  | 0.533 | 0.016 | [0.503, 0.565] | < 0.001 | | 0.518 | 0.015 | [0.489, 0.548] | < 0.001 | |
| Environmental Unpredictability | CON_DMN2 | 0.001 | 0.000 | [0.000, 0.001] | < 0.001 | | 0.035 | 0.008 | [0.021, 0.052] | < 0.001 | |
| Threat |  | 0.000 | 0.000 | [-0.001, 0.001] | 0.679 | | -0.004 | 0.010 | [-0.022, 0.016] | 0.679 | |
| Scanner Type |  | 0.008 | 0.007 | [0.001, 0.025] | 0.269 | | 0.091 | 0.053 | [0.013, 0.214] | 0.087 | |
| Framewise Displacement |  | 0.059 | 0.013 | [0.036, 0.084] | < 0.001 | | 0.157 | 0.024 | [0.108, 0.201] | < 0.001 | |
| Age2 |  | 0.000 | 0.000 | [-0.001, 0.000] | < 0.001 | | -0.049 | 0.013 | [-0.076, -0.025] | < 0.001 | |
| Race |  | 0.002 | 0.001 | [0.000, 0.003] | 0.025 | | 0.037 | 0.016 | [0.002, 0.063] | 0.021 | |
| Sex |  | -0.007 | 0.002 | [-0.011, -0.004] | < 0.001 | | -0.065 | 0.015 | [-0.094, -0.035] | < 0.001 | |
| CON_DMN0 |  | 0.431 | 0.019 | [0.397, 0.469] | < 0.001 | | 0.409 | 0.017 | [0.377, 0.445] | < 0.001 | |
| Environmental Unpredictability | CON_FPN2 | 0.000 | 0.000 | [0.000, 0.000] | 0.763 | | -0.003 | 0.011 | [-0.027, 0.018] | 0.760 | |
| Threat |  | 0.000 | 0.000 | [0.000, 0.001] | 0.227 | | 0.015 | 0.012 | [-0.010, 0.038] | 0.216 | |
| Scanner Type |  | 0.004 | 0.003 | [0.001, 0.012] | 0.208 | | 0.059 | 0.028 | [0.012, 0.118] | 0.038 | |
| Framewise Displacement |  | 0.017 | 0.006 | [0.006, 0.028] | 0.007 | | 0.055 | 0.017 | [0.020, 0.086] | 0.001 | |
| Age2 |  | 0.000 | 0.000 | [0.000, 0.000] | 0.005 | | -0.035 | 0.012 | [-0.059, -0.011] | 0.005 | |
| Race |  | 0.001 | 0.000 | [0.000, 0.002] | 0.093 | | 0.023 | 0.013 | [-0.003, 0.049] | 0.082 | |
| Sex |  | -0.006 | 0.001 | [-0.008, -0.004] | < 0.001 | | -0.066 | 0.010 | [-0.086, -0.047] | < 0.001 | |
| CON_FPN0 |  | 0.418 | 0.013 | [0.391, 0.441] | < 0.001 | | 0.407 | 0.013 | [0.379, 0.429] | < 0.001 | |
| Environmental Unpredictability | DMN_FPN2 | 0.000 | 0.000 | [0.000, 0.000] | 0.748 | | -0.005 | 0.015 | [-0.033, 0.027] | 0.746 | |
| Threat |  | 0.000 | 0.000 | [-0.001, 0.001] | 0.723 | | -0.004 | 0.012 | [-0.027, 0.018] | 0.724 | |
| Scanner Type |  | 0.003 | 0.003 | [0.000, 0.010] | 0.270 | | 0.052 | 0.031 | [-0.001, 0.113] | 0.092 | |
| Framewise Displacement |  | 0.028 | 0.006 | [0.017, 0.040] | < 0.001 | | 0.093 | 0.014 | [0.065, 0.121] | < 0.001 | |
| Age2 |  | 0.000 | 0.000 | [0.000, 0.000] | 0.001 | | 0.030 | 0.009 | [0.012, 0.047] | 0.001 | |
| Race |  | 0.001 | 0.001 | [0.000, 0.003] | 0.034 | | 0.044 | 0.019 | [0.009, 0.084] | 0.025 | |
| Sex |  | 0.001 | 0.001 | [0.000, 0.003] | 0.088 | | 0.015 | 0.009 | [-0.001, 0.032] | 0.086 | |
| DMN_FPN0 |  | 0.472 | 0.014 | [0.444, 0.497] | < 0.001 | | 0.450 | 0.013 | [0.427, 0.477] | < 0.001 | |

Note. CON = average correlations within the Cingulo-Opercular Network, DMN = average correlations within the Default Mode Network, FPN = average correlations within the Fronto-Parietal Network, CON_DMN = average correlations between Cingulo-Opercular Network and Default Mode Network, CON_FPN = average correlations between Cingulo-Opercular Network and Fronto-Parietal Network, DMN_FPN = average correlations between Default Mode Network and Fronto-Parietal Network. And the number following the abbreviation of the variable name represents the time of data collection, 0 = baseline, 2 = 2-year follow-up. Due to resampling in the cluster-robust standard error model, model fit indices (e.g., chi-square, CFI, and TLI) were not available with replicate weights. Based on the available indices (RMSEA = 0.000, SRMR = 0.000), the models fitted well. The reported *p* values were uncorrected.

Table S4. Moderating Effect of Neighborhood Educational Deprivation on the Association between Environmental Unpredictability and rsFC.

| Independent Variable and Covariates | Dependent Variable | Unstandardized | | | | Standardized | | | | |  |
| --- | --- | --- | --- | --- | --- | --- | --- | --- | --- | --- | --- |
|  |  | Coefficient | *SE* | *95% CI* | *p* | | Coefficient | *SE* | *95% CI* | *p* | |
| EU | CON2 | -0.017 | 0.012 | [-0.041, 0.006] | 0.156 | | -0.017 | 0.012 | [-0.041, 0.006] | 0.156 | |
| Threat |  | -0.011 | 0.007 | [-0.025, 0.002] | 0.116 | | -0.018 | 0.011 | [-0.039, 0.004] | 0.105 | |
| Scanner Type |  | -0.033 | 0.035 | [-0.097, 0.030] | 0.347 | | -0.022 | 0.019 | [-0.058, 0.017] | 0.260 | |
| Framewise Displacement |  | -0.606 | 0.108 | [-0.832, -0.412] | < 0.001 | | -0.091 | 0.012 | [-0.112, -0.064] | < 0.001 | |
| Age2 |  | 0.003 | 0.001 | [0.001, 0.005] | 0.011 | | 0.024 | 0.010 | [0.005, 0.043] | 0.011 | |
| Race |  | -0.023 | 0.009 | [-0.042, -0.007] | 0.011 | | -0.030 | 0.011 | [-0.055, -0.010] | 0.008 | |
| Sex |  | 0.075 | 0.029 | [0.012, 0.127] | 0.009 | | 0.037 | 0.015 | [0.007, 0.064] | 0.010 | |
| CON0 |  | 7.250 | 0.143 | [6.958, 7.513] | < 0.001 | | 0.508 | 0.011 | [0.485, 0.527] | < 0.001 | |
| ND_E |  | 0.020 | 0.013 | [-0.004, 0.045] | 0.130 | | 0.020 | 0.013 | [-0.004, 0.044] | 0.121 | |
| EU × ND_E |  | 0.009 | 0.012 | [-0.014, 0.032] | 0.426 | | 0.009 | 0.012 | [-0.013, 0.032] | 0.426 | |
| EU | DMN2 | -0.031 | 0.011 | [-0.054, -0.011] | 0.005 | | -0.031 | 0.011 | [-0.052, -0.011] | 0.003 | |
| Threat |  | -0.005 | 0.007 | [-0.018, 0.008] | 0.420 | | -0.009 | 0.011 | [-0.029, 0.013] | 0.418 | |
| Scanner Type |  | -0.144 | 0.108 | [-0.402, -0.040] | 0.182 | | -0.095 | 0.045 | [-0.206, -0.033] | 0.033 | |
| Framewise Displacement |  | -0.557 | 0.136 | [-0.820, -0.311] | < 0.001 | | -0.083 | 0.016 | [-0.111, -0.051] | < 0.001 | |
| Age2 |  | 0.002 | 0.001 | [-0.001, 0.005] | 0.151 | | 0.017 | 0.012 | [-0.004, 0.040] | 0.150 | |
| Race |  | -0.027 | 0.007 | [-0.043, -0.014] | < 0.001 | | -0.035 | 0.009 | [-0.055, -0.018] | < 0.001 | |
| Sex |  | 0.160 | 0.018 | [0.127, 0.194] | < 0.001 | | 0.080 | 0.009 | [0.063, 0.097] | < 0.001 | |
| DMN0 |  | 8.654 | 0.486 | [7.587, 9.367] | < 0.001 | | 0.497 | 0.028 | [0.435, 0.536] | < 0.001 | |
| ND_E |  | 0.036 | 0.016 | [0.006, 0.069] | 0.028 | | 0.036 | 0.016 | [0.006, 0.068] | 0.026 | |
| EU × ND_E |  | 0.034 | 0.013 | [0.010, 0.061] | 0.009 | | 0.034 | 0.012 | [0.011, 0.057] | 0.004 | |
| EU | FPN2 | 0.011 | 0.010 | [-0.014, 0.027] | 0.271 | | 0.011 | 0.010 | [-0.013, 0.028] | 0.274 | |
| Threat |  | -0.008 | 0.007 | [-0.023, 0.005] | 0.240 | | -0.014 | 0.012 | [-0.037, 0.008] | 0.243 | |
| Scanner Type |  | -0.031 | 0.049 | [-0.151, 0.034] | 0.525 | | -0.020 | 0.025 | [-0.071, 0.026] | 0.409 | |
| Framewise Displacement |  | -0.515 | 0.149 | [-0.773, -0.228] | 0.001 | | -0.077 | 0.018 | [-0.105, -0.039] | < 0.001 | |
| Age2 |  | 0.001 | 0.001 | [-0.002, 0.004] | 0.312 | | 0.011 | 0.011 | [-0.012, 0.033] | 0.313 | |
| Race |  | -0.013 | 0.008 | [-0.028, 0.001] | 0.099 | | -0.017 | 0.011 | [-0.040, 0.002] | 0.108 | |
| Sex |  | 0.119 | 0.026 | [0.069, 0.170] | < 0.001 | | 0.060 | 0.013 | [0.035, 0.084] | < 0.001 | |
| FPN0 |  | 9.223 | 0.278 | [8.710, 9.755] | < 0.001 | | 0.518 | 0.015 | [0.490, 0.548] | < 0.001 | |
| ND_E |  | -0.012 | 0.018 | [-0.043, 0.031] | 0.501 | | -0.012 | 0.018 | [-0.043, 0.030] | 0.499 | |
| EU × ND_E |  | 0.025 | 0.009 | [0.008, 0.045] | 0.006 | | 0.025 | 0.008 | [0.009, 0.041] | 0.003 | |
| EU | CON_DMN2 | 0.026 | 0.009 | [0.007, 0.043] | 0.006 | | 0.026 | 0.009 | [0.007, 0.044] | 0.007 | |
| Threat |  | -0.003 | 0.006 | [-0.014, 0.010] | 0.600 | | -0.005 | 0.010 | [-0.023, 0.015] | 0.601 | |
| Scanner Type |  | 0.137 | 0.124 | [0.017, 0.434] | 0.268 | | 0.091 | 0.503 | [0.015, 0.212] | 0.084 | |
| Framewise Displacement |  | 1.039 | 0.227 | [0.625, 1.477] | < 0.001 | | 0.155 | 0.024 | [0.106, 0.199] | < 0.001 | |
| Age2 |  | -0.006 | 0.002 | [-0.010, -0.003] | < 0.001 | | -0.050 | 0.013 | [-0.076, -0.025] | < 0.001 | |
| Race |  | 0.024 | 0.010 | [0.004, 0.044] | 0.015 | | 0.032 | 0.013 | [0.006, 0.056] | 0.011 | |
| Sex |  | -0.132 | 0.029 | [-0.188, -0.075] | < 0.001 | | -0.066 | 0.015 | [-0.095, -0.038] | < 0.001 | |
| CON_DMN0 |  | 7.618 | 0.321 | [7.026, 8.289] | < 0.001 | | 0.408 | 0.017 | [0.376, 0.442] | < 0.001 | |
| ND_E |  | -0.030 | 0.020 | [-0.070, 0.007] | 0.126 | | -0.030 | 0.020 | [-0.069, 0.006] | 0.121 | |
| EU × ND_E |  | -0.028 | 0.011 | [-0.051, -0.009] | 0.008 | | -0.028 | 0.010 | [-0.048, -0.009] | 0.004 | |
| EU | CON_FPN2 | -0.004 | 0.012 | [-0.028, 0.018] | 0.729 | | -0.004 | 0.012 | [-0.027, 0.019] | 0.726 | |
| Threat |  | 0.009 | 0.008 | [-0.006, 0.024] | 0.227 | | 0.015 | 0.012 | [-0.010, 0.037] | 0.217 | |
| Scanner Type |  | 0.089 | 0.072 | [0.015, 0.250] | 0.214 | | 0.059 | 0.029 | [0.011, 0.118] | 0.041 | |
| Framewise Displacement |  | 0.365 | 0.138 | [0.114, 0.625] | 0.008 | | 0.055 | 0.017 | [0.020, 0.086] | 0.002 | |
| Age2 |  | -0.004 | 0.002 | [-0.008, -0.001] | 0.005 | | -0.035 | 0.012 | [-0.059, -0.011] | 0.005 | |
| Race |  | 0.017 | 0.010 | [-0.001, 0.039] | 0.088 | | 0.023 | 0.013 | [-0.001, 0.050] | 0.077 | |
| Sex |  | -0.133 | 0.020 | [-0.174, -0.093] | < 0.001 | | -0.066 | 0.010 | [-0.086, -0.047] | < 0.001 | |
| CON_FPN0 |  | 9.150 | 0.275 | [8.568, 9.650] | < 0.001 | | 0.407 | 0.013 | [0.379, 0.429] | < 0.001 | |
| ND_E |  | -0.001 | 0.015 | [-0.031, 0.027] | 0.949 | | -0.001 | 0.015 | [-0.030, 0.027] | 0.949 | |
| EU × ND_E |  | -0.002 | 0.009 | [-0.019, 0.017] | 0.836 | | -0.002 | 0.009 | [-0.020, 0.016] | 0.835 | |
| EU | DMN_FPN2 | -0.008 | 0.015 | [-0.036, 0.022] | 0.602 | | -0.008 | 0.014 | [-0.035, 0.023] | 0.599 | |
| Threat |  | -0.003 | 0.008 | [-0.018, 0.012] | 0.709 | | -0.005 | 0.013 | [-0.029, 0.020] | 0.711 | |
| Scanner Type |  | 0.078 | 0.070 | [0.001, 0.227] | 0.264 | | 0.052 | 0.030 | [0.001, 0.112] | 0.084 | |
| Framewise Displacement |  | 0.614 | 0.132 | [0.390, 0.902] | < 0.001 | | 0.092 | 0.014 | [0.064, 0.120] | < 0.001 | |
| Age2 |  | 0.004 | 0.001 | [0.002, 0.006] | 0.001 | | 0.030 | 0.009 | [0.012, 0.047] | 0.001 | |
| Race |  | 0.030 | 0.015 | [0.004, 0.064] | 0.051 | | 0.040 | 0.019 | [0.006, 0.081] | 0.040 | |
| Sex |  | 0.029 | 0.017 | [-0.003, 0.063] | 0.095 | | 0.014 | 0.008 | [-0.001, 0.031] | 0.092 | |
| DMN_FPN0 |  | 10.585 | 0.311 | [9.926, 11.142] | < 0.001 | | 0.450 | 0.013 | [0.426, 0.476] | < 0.001 | |
| ND_E |  | -0.023 | 0.016 | [-0.061, 0.005] | 0.154 | | -0.023 | 0.016 | [-0.059, 0.005] | 0.143 | |
| EU × ND_E |  | 0.001 | 0.013 | [-0.023, 0.026] | 0.929 | | 0.001 | 0.013 | [-0.023, 0.026] | 0.928 | |

Note. EU = Environmental Unpredictability, ND_E = Neighborhood Deprivation in Education domain, CON = average correlations within the Cingulo-Opercular Network, DMN = average correlations within the Default Mode Network, FPN = average correlations within the Fronto-Parietal Network, CON_DMN = average correlations between Cingulo-Opercular Network and Default Mode Network, CON_FPN = average correlations between Cingulo-Opercular Network and Fronto-Parietal Network, DMN_FPN = average correlations between Default Mode Network and Fronto-Parietal Network. And the number following the abbreviation of the variable name represents the time of data collection, 0 = baseline, 2 = 2-year follow-up. Due to resampling in the cluster-robust standard error model, model fit indices (e.g., chi-square, CFI, and TLI) were not available with replicate weights. Based on the available indices (RMSEA = 0.000, SRMR = 0.000), the models fitted well. The reported *p* values were uncorrected.

Table S5. Moderating Effect of Neighborhood Health Deprivation on the Association between Environmental Unpredictability and rsFC.

| Independent Variable and Covariates | Dependent Variable | Unstandardized | | | | Standardized | | | | |  |
| --- | --- | --- | --- | --- | --- | --- | --- | --- | --- | --- | --- |
|  |  | Coefficient | *SE* | *95% CI* | *p* | | Coefficient | *SE* | *95% CI* | *p* | |
| EU | CON2 | -0.017 | 0.013 | [-0.042, 0.010] | 0.191 | | -0.017 | 0.013 | [-0.042, 0.010] | 0.197 | |
| Threat |  | -0.011 | 0.008 | [-0.027, 0.004] | 0.176 | | -0.017 | 0.013 | [-0.042, 0.007] | 0.177 | |
| Age2 |  | 0.003 | 0.002 | [0.000, 0.006] | 0.071 | | 0.022 | 0.012 | [-0.002, 0.047] | 0.072 | |
| Sex |  | 0.078 | 0.031 | [0.021, 0.143] | 0.011 | | 0.039 | 0.016 | [0.010, 0.072] | 0.013 | |
| Framewise Displacement |  | -0.589 | 0.089 | [-0.733, -0.386] | < 0.001 | | -0.088 | 0.015 | [-0.114, -0.055] | < 0.001 | |
| Scanner Type |  | -0.036 | 0.040 | [-0.140, 0.017] | 0.373 | | -0.024 | 0.021 | [-0.073, 0.011] | 0.269 | |
| Race |  | -0.018 | 0.009 | [-0.037, -0.001] | 0.054 | | -0.024 | 0.012 | [-0.048, -0.002] | 0.044 | |
| CON0 |  | 7.159 | 0.288 | [6.594, 7.649] | < 0.001 | | 0.502 | 0.021 | [0.461, 0.535] | < 0.001 | |
| ND_HE |  | 0.057 | 0.014 | [0.033, 0.087] | < 0.001 | | 0.057 | 0.015 | [0.030, 0.088] | < 0.001 | |
| EU × ND_HE |  | -0.007 | 0.010 | [-0.026, 0.013] | 0.492 | | -0.007 | 0.010 | [-0.028, 0.012] | 0.493 | |
| EU | DMN2 | -0.033 | 0.010 | [-0.057, -0.015] | 0.002 | | -0.033 | 0.010 | [-0.055, -0.016] | 0.001 | |
| Threat |  | -0.005 | 0.005 | [-0.015, 0.006] | 0.369 | | -0.008 | 0.008 | [-0.024, 0.009] | 0.371 | |
| Age2 |  | 0.002 | 0.002 | [-0.002, 0.006] | 0.427 | | 0.014 | 0.017 | [-0.019, 0.048] | 0.429 | |
| Sex |  | 0.163 | 0.020 | [0.125, 0.205] | < 0.001 | | 0.081 | 0.010 | [0.062, 0.102] | < 0.001 | |
| Framewise Displacement |  | -0.535 | 0.094 | [-0.725, -0.365] | < 0.001 | | -0.080 | 0.011 | [-0.100, -0.056] | < 0.001 | |
| Scanner Type |  | -0.146 | 0.091 | [-0.421, -0.036] | 0.110 | | -0.096 | 0.040 | [-0.193, -0.030] | 0.016 | |
| Race |  | -0.022 | 0.012 | [-0.051, 0.003] | 0.071 | | -0.029 | 0.016 | [-0.065, -0.004] | 0.068 | |
| DMN0 |  | 8.597 | 0.286 | [8.040, 9.083] | < 0.001 | | 0.494 | 0.016 | [0.464, 0.523] | < 0.001 | |
| ND_HE |  | 0.071 | 0.027 | [0.028, 0.124] | 0.009 | | 0.071 | 0.029 | [0.027, 0.139] | 0.015 | |
| EU × ND_HE |  | 0.016 | 0.014 | [-0.013, 0.040] | 0.240 | | 0.016 | 0.013 | [-0.013, 0.039] | 0.225 | |
| EU | FPN2 | 0.008 | 0.010 | [-0.013, 0.028] | 0.404 | | 0.008 | 0.010 | [-0.013, 0.028] | 0.404 | |
| Threat |  | -0.008 | 0.006 | [-0.020, 0.003] | 0.141 | | -0.013 | 0.009 | [-0.031, 0.005] | 0.142 | |
| Age2 |  | 0.001 | 0.002 | [-0.002, 0.005] | 0.394 | | 0.011 | 0.013 | [-0.013, 0.037] | 0.396 | |
| Sex |  | 0.120 | 0.025 | [0.071, 0.170] | < 0.001 | | 0.060 | 0.013 | [0.036, 0.085] | < 0.001 | |
| Framewise Displacement |  | -0.509 | 0.116 | [-0.716, -0.285] | < 0.001 | | -0.076 | 0.015 | [-0.103, -0.046] | < 0.001 | |
| Scanner Type |  | -0.031 | 0.072 | [-0.249, 0.054] | 0.665 | | -0.021 | 0.038 | [-0.111, 0.042] | 0.583 | |
| Race |  | -0.010 | 0.012 | [-0.033, 0.015] | 0.441 | | -0.013 | 0.017 | [-0.045, 0.019] | 0.444 | |
| FPN0 |  | 9.216 | 0.306 | [8.670, 9.791] | < 0.001 | | 0.517 | 0.013 | [0.494, 0.542] | < 0.001 | |
| ND_HE |  | 0.012 | 0.026 | [-0.030, 0.067] | 0.634 | | 0.012 | 0.026 | [-0.028, 0.070] | 0.632 | |
| EU × ND_HE |  | 0.001 | 0.013 | [-0.022, 0.026] | 0.932 | | 0.001 | 0.013 | [-0.023, 0.025] | 0.928 | |
| EU | CON_DMN2 | 0.025 | 0.009 | [0.009, 0.043] | 0.005 | | 0.025 | 0.009 | [0.008, 0.044] | 0.006 | |
| Threat |  | -0.004 | 0.005 | [-0.012, 0.008] | 0.403 | | -0.007 | 0.008 | [-0.020, 0.012] | 0.407 | |
| Age2 |  | -0.006 | 0.002 | [-0.009, -0.002] | 0.001 | | -0.046 | 0.014 | [-0.074, -0.017] | 0.001 | |
| Sex |  | -0.134 | 0.031 | [-0.195, -0.075] | < 0.001 | | -0.067 | 0.015 | [-0.097, -0.037] | < 0.001 | |
| Framewise Displacement |  | 1.007 | 0.150 | [0.718, 1.295] | < 0.001 | | 0.151 | 0.015 | [0.119, 0.179] | < 0.001 | |
| Scanner Type |  | 0.138 | 0.072 | [0.037, 0.322] | 0.057 | | 0.091 | 0.033 | [0.030, 0.161] | 0.006 | |
| Race |  | 0.017 | 0.012 | [-0.006, 0.039] | 0.143 | | 0.023 | 0.015 | [-0.007, 0.050] | 0.136 | |
| CON_DMN0 |  | 7.616 | 0.237 | [7.223, 8.168] | < 0.001 | | 0.408 | 0.013 | [0.384, 0.437] | < 0.001 | |
| ND_HE |  | -0.081 | 0.021 | [-0.126, -0.043] | < 0.001 | | -0.081 | 0.022 | [-0.127, -0.042] | < 0.001 | |
| EU × ND_HE |  | -0.012 | 0.011 | [-0.031, 0.011] | 0.267 | | -0.012 | 0.011 | [-0.031, 0.011] | 0.258 | |
| EU | CON_FPN2 | -0.006 | 0.010 | [-0.025, 0.016] | 0.538 | | -0.006 | 0.010 | [-0.024, 0.016] | 0.531 | |
| Threat |  | 0.009 | 0.007 | [-0.005, 0.024] | 0.235 | | 0.014 | 0.011 | [-0.008, 0.037] | 0.224 | |
| Age2 |  | -0.004 | 0.002 | [-0.007, -0.001] | 0.006 | | -0.034 | 0.012 | [-0.056, -0.009] | 0.006 | |
| Sex |  | -0.135 | 0.018 | [-0.170, -0.098] | < 0.001 | | -0.067 | 0.009 | [-0.085, -0.049] | < 0.001 | |
| Framewise Displacement |  | 0.350 | 0.105 | [0.157, 0.556] | 0.001 | | 0.052 | 0.014 | [0.025, 0.080] | < 0.001 | |
| Scanner Type |  | 0.089 | 0.030 | [0.021, 0.142] | 0.003 | | 0.059 | 0.021 | [0.016, 0.098] | 0.004 | |
| Race |  | 0.014 | 0.009 | [-0.005, 0.031] | 0.149 | | 0.018 | 0.012 | [-0.007, 0.041] | 0.141 | |
| CON_FPN0 |  | 9.143 | 0.345 | [8.449, 9.772] | < 0.001 | | 0.407 | 0.013 | [0.380, 0.431] | < 0.001 | |
| ND_HE |  | -0.028 | 0.022 | [-0.073, 0.015] | 0.216 | | -0.028 | 0.022 | [-0.075, 0.013] | 0.219 | |
| EU × ND_HE |  | -0.003 | 0.011 | [-0.026, 0.016] | 0.808 | | -0.003 | 0.011 | [-0.026, 0.017] | 0.805 | |
| EU | DMN_FPN2 | -0.007 | 0.014 | [-0.035, 0.018] | 0.596 | | -0.007 | 0.013 | [-0.034, 0.019] | 0.590 | |
| Threat |  | -0.003 | 0.007 | [-0.017, 0.011] | 0.661 | | -0.005 | 0.012 | [-0.028, 0.017] | 0.663 | |
| Age2 |  | 0.004 | 0.001 | [0.002, 0.006] | < 0.001 | | 0.031 | 0.008 | [0.016, 0.046] | < 0.001 | |
| Sex |  | 0.028 | 0.016 | [0.000, 0.062] | 0.074 | | 0.014 | 0.008 | [0.000, 0.031] | 0.074 | |
| Framewise Displacement |  | 0.607 | 0.085 | [0.455, 0.785] | < 0.001 | | 0.091 | 0.010 | [0.069, 0.110] | < 0.001 | |
| Scanner Type |  | 0.078 | 0.041 | [0.002, 0.159] | 0.054 | | 0.052 | 0.024 | [0.005, 0.097] | 0.028 | |
| Race |  | 0.030 | 0.012 | [0.009, 0.058] | 0.016 | | 0.039 | 0.015 | [0.013, 0.074] | 0.011 | |
| DMN_FPN0 |  | 10.599 | 0.384 | [9.925, 11.422] | < 0.001 | | 0.451 | 0.013 | [0.427, 0.476] | < 0.001 | |
| ND_HE |  | -0.025 | 0.021 | [-0.073, 0.010] | 0.231 | | -0.025 | 0.021 | [-0.076, 0.009] | 0.237 | |
| EU × ND_HE |  | 0.002 | 0.011 | [-0.019, 0.023] | 0.815 | | 0.003 | 0.011 | [-0.021, 0.022] | 0.811 | |

Note. EU = Environmental Unpredictability, ND_HE = Neighborhood Deprivation in Health and Environment domain, CON = average correlations within the Cingulo-Opercular Network, DMN = average correlations within the Default Mode Network, FPN = average correlations within the Fronto-Parietal Network, CON_DMN = average correlations between Cingulo-Opercular Network and Default Mode Network, CON_FPN = average correlations between Cingulo-Opercular Network and Fronto-Parietal Network, DMN_FPN = average correlations between Default Mode Network and Fronto-Parietal Network. And the number following the abbreviation of the variable name represents the time of data collection, 0 = baseline, 2 = 2-year follow-up. Due to resampling in the cluster-robust standard error model, model fit indices (e.g., chi-square, CFI, and TLI) were not available with replicate weights. Based on the available indices (RMSEA = 0.000, SRMR = 0.000), the models fitted well. The reported *p* values were uncorrected.

Table S6. Moderating Effect of Neighborhood Socioeconomic Deprivation on the Association between Environmental Unpredictability and rsFC.

| Independent Variable and Covariates | Dependent Variable | Unstandardized | | | | Standardized | | | | |  |
| --- | --- | --- | --- | --- | --- | --- | --- | --- | --- | --- | --- |
|  |  | Coefficient | *SE* | *95% CI* | *p* | | Coefficient | *SE* | *95% CI* | *p* | |
| EU | CON2 | -0.014 | 0.014 | [-0.040, 0.016] | 0.327 | | -0.014 | 0.014 | [-0.040, 0.016] | 0.331 | |
| Threat |  | -0.010 | 0.008 | [-0.026, 0.004] | 0.183 | | -0.016 | 0.012 | [-0.041, 0.007] | 0.184 | |
| Age2 |  | 0.003 | 0.002 | [0.000, 0.006] | 0.067 | | 0.023 | 0.012 | [-0.001, 0.048] | 0.067 | |
| Sex |  | 0.077 | 0.031 | [0.019, 0.142] | 0.013 | | 0.038 | 0.016 | [0.009, 0.071] | 0.015 | |
| Framewise Displacement |  | -0.600 | 0.088 | [-0.740, -0.398] | < 0.001 | | -0.090 | 0.015 | [-0.115, -0.057] | < 0.001 | |
| Scanner Type |  | -0.033 | 0.043 | [-0.152, 0.025] | 0.446 | | -0.022 | 0.023 | [-0.076, 0.017] | 0.347 | |
| Race |  | -0.018 | 0.009 | [-0.036, -0.002] | 0.038 | | -0.024 | 0.011 | [-0.047, -0.003] | 0.031 | |
| CON0 |  | 7.174 | 0.276 | [6.614, 7.618] | < 0.001 | | 0.503 | 0.020 | [0.464, 0.536] | < 0.001 | |
| ND_SE |  | 0.046 | 0.015 | [0.015, 0.072] | 0.002 | | 0.046 | 0.015 | [0.015, 0.073] | 0.002 | |
| EU × ND_SE |  | 0.010 | 0.011 | [-0.010, 0.032] | 0.337 | | 0.011 | 0.012 | [-0.011, 0.034] | 0.336 | |
| EU | DMN2 | -0.033 | 0.010 | [-0.056, -0.017] | 0.001 | | -0.033 | 0.009 | [-0.053, -0.017] | < 0.001 | |
| Threat |  | -0.005 | 0.005 | [-0.015, 0.006] | 0.386 | | -0.007 | 0.008 | [-0.024, 0.009] | 0.387 | |
| Age2 |  | 0.002 | 0.002 | [-0.002, 0.006] | 0.398 | | 0.015 | 0.018 | [-0.019, 0.051] | 0.399 | |
| Sex |  | 0.160 | 0.020 | [0.122, 0.200] | < 0.001 | | 0.080 | 0.010 | [0.061, 0.100] | < 0.001 | |
| Framewise Displacement |  | -0.556 | 0.094 | [-0.748, -0.383] | < 0.001 | | -0.083 | 0.011 | [-0.104, -0.060] | < 0.001 | |
| Scanner Type |  | -0.143 | 0.097 | [-0.438, -0.028] | 0.139 | | -0.095 | 0.044 | [-0.204, -0.025] | 0.029 | |
| Race |  | -0.024 | 0.012 | [-0.052, -0.004] | 0.059 | | -0.031 | 0.017 | [-0.068, -0.005] | 0.059 | |
| DMN0 |  | 8.623 | 0.283 | [8.080, 9.098] | < 0.001 | | 0.496 | 0.015 | [0.466, 0.523] | < 0.001 | |
| ND_SE |  | 0.046 | 0.018 | [0.011, 0.082] | 0.013 | | 0.046 | 0.018 | [0.011, 0.079] | 0.010 | |
| EU × ND_SE |  | 0.020 | 0.014 | [-0.005, 0.048] | 0.135 | | 0.022 | 0.015 | [-0.005, 0.054] | 0.147 | |
| EU | FPN2 | 0.008 | 0.011 | [-0.015, 0.028] | 0.432 | | 0.008 | 0.011 | [-0.015, 0.028] | 0.432 | |
| Threat |  | -0.009 | 0.006 | [-0.020, 0.003] | 0.125 | | -0.014 | 0.009 | [-0.032, 0.004] | 0.127 | |
| Age2 |  | 0.001 | 0.002 | [-0.002, 0.005] | 0.360 | | 0.012 | 0.013 | [-0.012, 0.038] | 0.362 | |
| Sex |  | 0.119 | 0.025 | [0.070, 0.168] | < 0.001 | | 0.060 | 0.013 | [0.035, 0.084] | < 0.001 | |
| Framewise Displacement |  | -0.521 | 0.116 | [-0.731, -0.298] | < 0.001 | | -0.078 | 0.015 | [-0.105, -0.048] | < 0.001 | |
| Scanner Type |  | -0.032 | 0.072 | [-0.252, 0.053] | 0.658 | | -0.021 | 0.038 | [-0.113, 0.040] | 0.576 | |
| Race |  | -0.014 | 0.012 | [-0.038, 0.009] | 0.274 | | -0.018 | 0.017 | [-0.053, 0.011] | 0.281 | |
| FPN0 |  | 9.216 | 0.303 | [8.675, 9.784] | < 0.001 | | 0.517 | 0.013 | [0.493, 0.542] | < 0.001 | |
| ND_SE |  | -0.014 | 0.018 | [-0.049, 0.021] | 0.436 | | -0.014 | 0.018 | [-0.049, 0.021] | 0.435 | |
| EU × ND_SE |  | 0.013 | 0.012 | [-0.012, 0.035] | 0.260 | | 0.014 | 0.013 | [-0.013, 0.037] | 0.255 | |
| EU | CON_DMN2 | 0.026 | 0.009 | [0.009, 0.042] | 0.003 | | 0.026 | 0.009 | [0.009, 0.042] | 0.004 | |
| Threat |  | -0.004 | 0.005 | [-0.012, 0.007] | 0.369 | | -0.007 | 0.008 | [-0.020, 0.011] | 0.374 | |
| Age2 |  | -0.006 | 0.002 | [-0.010, -0.002] | 0.001 | | -0.048 | 0.015 | [-0.077, -0.018] | 0.001 | |
| Sex |  | -0.133 | 0.031 | [-0.194, -0.072] | < 0.001 | | -0.066 | 0.015 | [-0.097, -0.036] | < 0.001 | |
| Framewise Displacement |  | 1.031 | 0.149 | [0.738, 1.312] | < 0.001 | | 0.154 | 0.015 | [0.124, 0.183] | < 0.001 | |
| Scanner Type |  | 0.135 | 0.077 | [0.033, 0.349] | 0.078 | | 0.089 | 0.035 | [0.027, 0.168] | 0.011 | |
| Race |  | 0.018 | 0.012 | [-0.006, 0.039] | 0.132 | | 0.024 | 0.016 | [-0.009, 0.051] | 0.128 | |
| CON_DMN0 |  | 7.585 | 0.232 | [7.194, 8.137] | < 0.001 | | 0.406 | 0.013 | [0.382, 0.436] | < 0.001 | |
| ND_SE |  | -0.059 | 0.014 | [-0.085, -0.033] | < 0.001 | | -0.059 | 0.014 | [-0.087, -0.033] | < 0.001 | |
| EU × ND_SE |  | -0.012 | 0.009 | [-0.030, 0.005] | 0.192 | | -0.013 | 0.010 | [-0.033, 0.005] | 0.193 | |
| EU | CON_FPN2 | -0.005 | 0.010 | [-0.024, 0.016] | 0.613 | | -0.005 | 0.010 | [-0.023, 0.016] | 0.607 | |
| Threat |  | 0.009 | 0.007 | [-0.005, 0.024] | 0.224 | | 0.014 | 0.011 | [-0.008, 0.036] | 0.213 | |
| Age2 |  | -0.004 | 0.002 | [-0.007, -0.001] | 0.006 | | -0.035 | 0.012 | [-0.057, -0.009] | 0.005 | |
| Sex |  | -0.134 | 0.019 | [-0.169, -0.096] | < 0.001 | | -0.067 | 0.009 | [-0.085, -0.048] | < 0.001 | |
| Framewise Displacement |  | 0.359 | 0.107 | [0.159, 0.567] | 0.001 | | 0.054 | 0.014 | [0.025, 0.081] | < 0.001 | |
| Scanner Type |  | 0.088 | 0.027 | [0.027, 0.136] | 0.001 | | 0.059 | 0.018 | [0.021, 0.092] | 0.001 | |
| Race |  | 0.014 | 0.010 | [-0.005, 0.033] | 0.136 | | 0.019 | 0.012 | [-0.006, 0.042] | 0.127 | |
| CON_FPN0 |  | 9.143 | 0.337 | [8.452, 9.759] | < 0.001 | | 0.407 | 0.013 | [0.380, 0.430] | < 0.001 | |
| ND_SE |  | -0.017 | 0.015 | [-0.046, 0.013] | 0.249 | | -0.017 | 0.015 | [-0.048, 0.013] | 0.253 | |
| EU × ND_SE |  | 0.003 | 0.008 | [-0.014, 0.017] | 0.749 | | 0.003 | 0.009 | [-0.015, 0.018] | 0.749 | |
| EU | DMN_FPN2 | -0.007 | 0.013 | [-0.034, 0.018] | 0.618 | | -0.007 | 0.013 | [-0.033, 0.019] | 0.612 | |
| Threat |  | -0.003 | 0.007 | [-0.017, 0.011] | 0.654 | | -0.005 | 0.012 | [-0.028, 0.017] | 0.656 | |
| Age2 |  | 0.004 | 0.001 | [0.002, 0.006] | < 0.001 | | 0.031 | 0.008 | [0.015, 0.046] | < 0.001 | |
| Sex |  | 0.029 | 0.016 | [0.000, 0.063] | 0.074 | | 0.014 | 0.008 | [0.000, 0.031] | 0.074 | |
| Framewise Displacement |  | 0.608 | 0.084 | [0.457, 0.786] | < 0.001 | | 0.091 | 0.011 | [0.070, 0.110] | < 0.001 | |
| Scanner Type |  | 0.077 | 0.042 | [0.003, 0.164] | 0.069 | | 0.051 | 0.024 | [0.004, 0.097] | 0.037 | |
| Race |  | 0.028 | 0.011 | [0.008, 0.053] | 0.016 | | 0.037 | 0.015 | [0.010, 0.068] | 0.012 | |
| DMN_FPN0 |  | 10.588 | 0.383 | [9.920, 11.408] | < 0.001 | | 0.450 | 0.013 | [0.427, 0.476] | < 0.001 | |
| ND_SE |  | -0.032 | 0.016 | [-0.067, -0.003] | 0.050 | | -0.032 | 0.016 | [-0.065, -0.003] | 0.042 | |
| EU × ND_SE |  | 0.010 | 0.011 | [-0.013, 0.030] | 0.352 | | 0.011 | 0.012 | [-0.014, 0.034] | 0.357 | |

Note. EU = Environmental Unpredictability, ND_SE = Neighborhood Deprivation in Social and Economic domain, CON = average correlations within the Cingulo-Opercular Network, DMN = average correlations within the Default Mode Network, FPN = average correlations within the Fronto-Parietal Network, CON_DMN = average correlations between Cingulo-Opercular Network and Default Mode Network, CON_FPN = average correlations between Cingulo-Opercular Network and Fronto-Parietal Network, DMN_FPN = average correlations between Default Mode Network and Fronto-Parietal Network. And the number following the abbreviation of the variable name represents the time of data collection, 0 = baseline, 2 = 2-year follow-up. Due to resampling in the cluster-robust standard error model, model fit indices (e.g., chi-square, CFI, and TLI) were not available with replicate weights. Based on the available indices (RMSEA = 0.000, SRMR = 0.000), the models fitted well. The reported *p* values were uncorrected.

Table S7. Moderating Effect of Overall Neighborhood Deprivation on the Association between Environmental Unpredictability and rsFC.

| Independent Variable and Covariates | Dependent Variable | Unstandardized | | | | Standardized | | | | |  |
| --- | --- | --- | --- | --- | --- | --- | --- | --- | --- | --- | --- |
|  |  | Coefficient | *SE* | *95% CI* | *p* | | Coefficient | *SE* | *95% CI* | *p* | |
| EU | CON2 | -0.014 | 0.014 | [-0.040, 0.016] | 0.334 | | -0.014 | 0.014 | [-0.040, 0.017] | 0.338 | |
| Threat |  | -0.010 | 0.008 | [-0.026, 0.004] | 0.176 | | -0.017 | 0.012 | [-0.042, 0.007] | 0.176 | |
| Age2 |  | 0.003 | 0.002 | [0.000, 0.006] | 0.064 | | 0.023 | 0.012 | [-0.001, 0.048] | 0.064 | |
| Sex |  | 0.077 | 0.031 | [0.019, 0.142] | 0.013 | | 0.038 | 0.016 | [0.009, 0.071] | 0.015 | |
| Framewise Displacement |  | -0.598 | 0.087 | [-0.738, -0.396] | < 0.001 | | -0.089 | 0.015 | [-0.115, -0.057] | < 0.001 | |
| Scanner Type |  | -0.033 | 0.042 | [-0.150, 0.023] | 0.431 | | -0.022 | 0.023 | [-0.076, 0.016] | 0.331 | |
| Race |  | -0.018 | 0.008 | [-0.037, -0.002] | 0.043 | | -0.024 | 0.012 | [-0.048, -0.002] | 0.035 | |
| CON0 |  | 7.177 | 0.280 | [6.611, 7.632] | < 0.001 | | 0.503 | 0.020 | [0.463, 0.536] | < 0.001 | |
| ND |  | 0.045 | 0.140 | [0.016, 0.070] | 0.002 | | 0.045 | 0.015 | [0.016, 0.072] | 0.002 | |
| EU × ND |  | 0.009 | 0.010 | [-0.011, 0.031] | 0.396 | | 0.009 | 0.011 | [-0.012, 0.032] | 0.394 | |
| EU | DMN2 | -0.031 | 0.010 | [-0.053, -0.014] | 0.002 | | -0.031 | 0.009 | [-0.051, -0.015] | 0.001 | |
| Threat |  | -0.005 | 0.005 | [-0.015, 0.006] | 0.385 | | -0.007 | 0.008 | [-0.024, 0.009] | 0.387 | |
| Age2 |  | 0.002 | 0.002 | [-0.002, 0.006] | 0.390 | | 0.015 | 0.018 | [-0.019, 0.051] | 0.391 | |
| Sex |  | 0.161 | 0.020 | [0.123, 0.202] | < 0.001 | | 0.080 | 0.010 | [0.061, 0.101] | < 0.001 | |
| Framewise Displacement |  | -0.551 | 0.093 | [-0.741, -0.382] | < 0.001 | | -0.082 | 0.011 | [-0.103, -0.059] | < 0.001 | |
| Scanner Type |  | -0.144 | 0.096 | [-0.433, -0.030] | 0.132 | | -0.095 | 0.043 | [-0.202, -0.026] | 0.026 | |
| Race |  | -0.023 | 0.012 | [-0.051, -0.004] | 0.061 | | -0.030 | 0.016 | [-0.067, -0.005] | 0.060 | |
| DMN0 |  | 8.614 | 0.286 | [8.064, 9.096] | < 0.001 | | 0.495 | 0.016 | [0.465, 0.523] | < 0.001 | |
| ND |  | 0.051 | 0.020 | [0.015, 0.093] | 0.012 | | 0.051 | 0.020 | [0.016, 0.093] | 0.011 | |
| EU × ND |  | 0.024 | 0.014 | [-0.002, 0.052] | 0.083 | | 0.026 | 0.015 | [-0.002, 0.057] | 0.090 | |
| EU | FPN2 | 0.009 | 0.011 | [-0.014, 0.029] | 0.377 | | 0.009 | 0.011 | [-0.013, 0.029] | 0.377 | |
| Threat |  | -0.009 | 0.006 | [-0.020, 0.003] | 0.126 | | -0.014 | 0.009 | [-0.032, 0.004] | 0.128 | |
| Age2 |  | 0.001 | 0.002 | [-0.002, 0.005] | 0.367 | | 0.012 | 0.013 | [-0.012, 0.037] | 0.368 | |
| Sex |  | 0.119 | 0.025 | [0.071, 0.168] | < 0.001 | | 0.060 | 0.013 | [0.035, 0.084] | < 0.001 | |
| Framewise Displacement |  | -0.519 | 0.116 | [-0.727, -0.296] | < 0.001 | | -0.078 | 0.015 | [-0.105, -0.048] | < 0.001 | |
| Scanner Type |  | -0.031 | 0.072 | [-0.253, 0.054] | 0.663 | | -0.021 | 0.038 | [-0.112, 0.041] | 0.583 | |
| Race |  | -0.013 | 0.012 | [-0.037, 0.009] | 0.269 | | -0.018 | 0.016 | [-0.051, 0.011] | 0.277 | |
| FPN0 |  | 9.220 | 0.303 | [8.678, 9.788] | < 0.001 | | 0.518 | 0.013 | [0.494, 0.542] | < 0.001 | |
| ND |  | -0.012 | 0.020 | [-0.051, 0.029] | 0.569 | | -0.012 | 0.020 | [-0.050, 0.028] | 0.567 | |
| EU × ND |  | 0.016 | 0.012 | [-0.009, 0.038] | 0.190 | | 0.017 | 0.012 | [-0.010, 0.040] | 0.181 | |
| EU | CON_DMN2 | 0.024 | 0.009 | [0.007, 0.041] | 0.007 | | 0.024 | 0.009 | [0.007, 0.041] | 0.008 | |
| Threat |  | -0.004 | 0.005 | [-0.012, 0.007] | 0.374 | | -0.007 | 0.008 | [-0.020, 0.011] | 0.379 | |
| Age2 |  | -0.006 | 0.002 | [-0.010, -0.002] | 0.001 | | -0.048 | 0.015 | [-0.077, -0.018] | 0.001 | |
| Sex |  | -0.133 | 0.031 | [-0.195, -0.073] | < 0.001 | | -0.066 | 0.015 | [-0.097, -0.036] | < 0.001 | |
| Framewise Displacement |  | 1.028 | 0.149 | [0.736, 1.309] | < 0.001 | | 0.154 | 0.015 | [0.123, 0.182] | < 0.001 | |
| Scanner Type |  | 0.136 | 0.076 | [0.035, 0.344] | 0.072 | | 0.090 | 0.035 | [0.028, 0.167] | 0.009 | |
| Race |  | 0.018 | 0.012 | [-0.006, 0.040] | 0.132 | | 0.024 | 0.016 | [-0.008, 0.052] | 0.126 | |
| CON_DMN0 |  | 7.587 | 0.233 | [7.196, 8.148] | < 0.001 | | 0.406 | 0.013 | [0.382, 0.436] | < 0.001 | |
| ND |  | -0.060 | 0.015 | [-0.091, -0.032] | < 0.001 | | -0.060 | 0.016 | [-0.090, -0.031] | < 0.001 | |
| EU × ND |  | -0.017 | 0.009 | [-0.036, 0.000] | 0.064 | | -0.018 | 0.009 | [-0.037, 0.000] | 0.062 | |
| EU | CON_FPN2 | -0.005 | 0.010 | [-0.024, 0.016] | 0.596 | | -0.005 | 0.010 | [-0.023, 0.016] | 0.590 | |
| Threat |  | 0.009 | 0.007 | [-0.005, 0.024] | 0.221 | | 0.014 | 0.011 | [-0.008, 0.036] | 0.210 | |
| Age2 |  | -0.004 | 0.002 | [-0.007, -0.001] | 0.006 | | -0.035 | 0.012 | [-0.057, -0.010] | 0.005 | |
| Sex |  | -0.134 | 0.018 | [-0.169, -0.096] | < 0.001 | | -0.067 | 0.009 | [-0.085, -0.048] | < 0.001 | |
| Framewise Displacement |  | 0.359 | 0.107 | [0.159, 0.566] | 0.001 | | 0.054 | 0.014 | [0.025, 0.081] | < 0.001 | |
| Scanner Type |  | 0.089 | 0.028 | [0.027, 0.136] | 0.001 | | 0.059 | 0.019 | [0.020, 0.093] | 0.002 | |
| Race |  | 0.015 | 0.010 | [-0.004, 0.033] | 0.129 | | 0.019 | 0.012 | [-0.005, 0.043] | 0.121 | |
| CON_FPN0 |  | 9.144 | 0.339 | [8.452, 9.763] | < 0.001 | | 0.407 | 0.013 | [0.380, 0.430] | < 0.001 | |
| ND |  | -0.016 | 0.017 | [-0.049, 0.017] | 0.332 | | -0.016 | 0.017 | [-0.050, 0.017] | 0.334 | |
| EU × ND |  | 0.001 | 0.008 | [-0.017, 0.016] | 0.910 | | 0.001 | 0.009 | [-0.019, 0.016] | 0.909 | |
| EU | DMN_FPN2 | -0.007 | 0.014 | [-0.036, 0.018] | 0.590 | | -0.007 | 0.013 | [-0.034, 0.019] | 0.583 | |
| Threat |  | -0.003 | 0.007 | [-0.017, 0.011] | 0.651 | | -0.005 | 0.012 | [-0.028, 0.017] | 0.654 | |
| Age2 |  | 0.004 | 0.001 | [0.002, 0.006] | < 0.001 | | 0.030 | 0.008 | [0.015, 0.046] | < 0.001 | |
| Sex |  | 0.028 | 0.016 | [0.000, 0.063] | 0.075 | | 0.014 | 0.008 | [0.000, 0.031] | 0.075 | |
| Framewise Displacement |  | 0.608 | 0.084 | [0.456, 0.785] | < 0.001 | | 0.091 | 0.011 | [0.070, 0.110] | < 0.001 | |
| Scanner Type |  | 0.077 | 0.041 | [0.004, 0.162] | 0.062 | | 0.051 | 0.024 | [0.006, 0.097] | 0.032 | |
| Race |  | 0.028 | 0.012 | [0.008, 0.055] | 0.018 | | 0.037 | 0.015 | [0.010, 0.069] | 0.013 | |
| DMN_FPN0 |  | 10.588 | 0.383 | [9.921, 11.407] | < 0.001 | | 0.450 | 0.013 | [0.427, 0.476] | < 0.001 | |
| ND |  | -0.032 | 0.018 | [-0.072, -0.003] | 0.066 | | -0.032 | 0.017 | [-0.070, -0.004] | 0.058 | |
| EU × ND |  | 0.008 | 0.010 | [-0.013, 0.028] | 0.436 | | 0.009 | 0.011 | [-0.014, 0.029] | 0.436 | |

Note. EU = Environmental Unpredictability, ND = Overall Neighborhood Deprivation, CON = average correlations within the Cingulo-Opercular Network, DMN = average correlations within the Default Mode Network, FPN = average correlations within the Fronto-Parietal Network, CON_DMN = average correlations between Cingulo-Opercular Network and Default Mode Network, CON_FPN = average correlations between Cingulo-Opercular Network and Fronto-Parietal Network, DMN_FPN = average correlations between Default Mode Network and Fronto-Parietal Network. And the number following the abbreviation of the variable name represents the time of data collection, 0 = baseline, 2 = 2-year follow-up. Due to resampling in the cluster-robust standard error model, model fit indices (e.g., chi-square, CFI, and TLI) were not available with replicate weights. Based on the available indices (RMSEA = 0.000, SRMR = 0.000), the models fitted well. The reported *p* values were uncorrected.

Table S8. Moderating Effect of Sex on the Association between Environmental Unpredictability and rsFC.

| Independent Variable and Covariates | Dependent Variable | Unstandardized | | | | Standardized | | | | |  |
| --- | --- | --- | --- | --- | --- | --- | --- | --- | --- | --- | --- |
|  |  | Coefficient | *SE* | *95% CI* | *p* | | Coefficient | *SE* | *95% CI* | *p* | |
| EU | CON2 | -0.004 | 0.019 | [-0.040, 0.035] | 0.856 | | -0.004 | 0.019 | [-0.040, 0.035] | 0.857 | |
| Threat |  | -0.012 | 0.008 | [-0.028, 0.003] | 0.117 | | -0.019 | 0.012 | [-0.044, 0.004] | 0.118 | |
| Age2 |  | 0.003 | 0.002 | [0.000, 0.006] | 0.065 | | 0.024 | 0.013 | [-0.001, 0.051] | 0.065 | |
| Sex |  | 0.073 | 0.031 | [0.015, 0.139] | 0.019 | | 0.037 | 0.016 | [0.007, 0.070] | 0.021 | |
| Framewise Displacement |  | -0.611 | 0.087 | [-0.751, -0.412] | < 0.001 | | -0.091 | 0.015 | [-0.116, -0.059] | < 0.001 | |
| Scanner Type |  | -0.033 | 0.044 | [-0.148, 0.030] | 0.458 | | -0.022 | 0.024 | [-0.076, 0.020] | 0.368 | |
| Race |  | -0.025 | 0.010 | [-0.045, -0.007] | 0.008 | | -0.034 | 0.012 | [-0.057, -0.011] | 0.005 | |
| CON0 |  | 7.280 | 0.291 | [6.690, 7.742] | < 0.001 | | 0.510 | 0.021 | [0.470, 0.543] | < 0.001 | |
| EU × Sex |  | -0.034 | 0.029 | [-0.086, 0.026] | 0.240 | | -0.025 | 0.021 | [-0.063, 0.019] | 0.249 | |
| EU | DMN2 | -0.042 | 0.017 | [-0.079, -0.012] | 0.013 | | -0.042 | 0.017 | [-0.078, -0.012] | 0.012 | |
| Threat |  | -0.006 | 0.006 | [-0.017, 0.005] | 0.275 | | -0.010 | 0.009 | [-0.027, 0.008] | 0.278 | |
| Age2 |  | 0.002 | 0.002 | [-0.002, 0.007] | 0.373 | | 0.016 | 0.018 | [-0.018, 0.053] | 0.374 | |
| Sex |  | 0.157 | 0.020 | [0.119, 0.198] | < 0.001 | | 0.078 | 0.010 | [0.059, 0.099] | < 0.001 | |
| Framewise Displacement |  | -0.571 | 0.098 | [-0.772, -0.393] | < 0.001 | | -0.085 | 0.011 | [-0.106, -0.062] | < 0.001 | |
| Scanner Type |  | -0.144 | 0.097 | [-0.434, -0.026] | 0.140 | | -0.095 | 0.044 | [-0.204, -0.024] | 0.031 | |
| Race |  | -0.031 | 0.013 | [-0.060, -0.009] | 0.022 | | -0.041 | 0.018 | [-0.079, -0.012] | 0.022 | |
| DMN0 |  | 8.700 | 0.277 | [8.165, 9.163] | < 0.001 | | 0.500 | 0.05 | [0.472, 0.527] | < 0.001 | |
| EU ×Sex |  | 0.001 | 0.024 | [-0.044, 0.049] | 0.979 | | 0.000 | 0.017 | [-0.031, 0.035] | 0.979 | |
| EU | FPN2 | 0.015 | 0.015 | [-0.015, 0.043] | 0.304 | | 0.015 | 0.015 | [-0.014, 0.044] | 0.310 | |
| Threat |  | -0.009 | 0.006 | [-0.020, 0.003] | 0.130 | | -0.014 | 0.009 | [-0.033, 0.004] | 0.132 | |
| Age2 |  | 0.001 | 0.002 | [-0.002, 0.005] | 0.377 | | 0.011 | 0.013 | [-0.013, 0.037] | 0.379 | |
| Sex |  | 0.119 | 0.025 | [0.071, 0.168] | < 0.001 | | 0.060 | 0.013 | [0.035, 0.084] | < 0.001 | |
| Framewise Displacement |  | -0.515 | 0.115 | [-0.720, -0.292] | < 0.001 | | -0.077 | 0.015 | [-0.104, -0.047] | < 0.001 | |
| Scanner Type |  | -0.031 | 0.072 | [-0.251, 0.053] | 0.664 | | -0.021 | 0.038 | [-0.111, 0.040] | 0.583 | |
| Race |  | -0.011 | 0.014 | [-0.038, 0.015] | 0.426 | | -0.015 | 0.019 | [-0.054, 0.019] | 0.431 | |
| FPN0 |  | 9.226 | 0.301 | [8.679, 9.787] | < 0.001 | | 0.518 | 0.013 | [0.494, 0.543] | < 0.001 | |
| EU ×Sex |  | -0.015 | 0.020 | [-0.058, 0.021] | 0.450 | | -0.011 | 0.015 | [-0.044, 0.014] | 0.458 | |
| EU | CON_DMN2 | 0.018 | 0.021 | [-0.023, 0.052] | 0.384 | | 0.018 | 0.021 | [-0.023, 0.058] | 0.385 | |
| Threat |  | -0.002 | 0.005 | [-0.010, 0.007] | 0.644 | | -0.003 | 0.007 | [-0.016, 0.013] | 0.645 | |
| Age2 |  | -0.006 | 0.002 | [-0.010, -0.003] | 0.002 | | -0.049 | 0.015 | [-0.079, -0.018] | 0.002 | |
| Sex |  | -0.129 | 0.032 | [-0.192, -0.078] | < 0.001 | | -0.065 | 0.016 | [-0.096, -0.034] | < 0.001 | |
| Framewise Displacement |  | 1.051 | 0.153 | [0.753, 1.302] | < 0.001 | | 0.157 | 0.015 | [0.126, 0.186] | < 0.001 | |
| Scanner Type |  | 0.137 | 0.077 | [0.032, 0.302] | 0.076 | | 0.091 | 0.036 | [0.027, 0.170] | 0.011 | |
| Race |  | 0.028 | 0.013 | [0.002, 0.048] | 0.036 | | 0.037 | 0.017 | [0.003, 0.066] | 0.032 | |
| CON_DMN0 |  | 7.646 | 0.237 | [7.246, 8.101] | < 0.001 | | 0.409 | 0.014 | [0.385, 0.440] | < 0.001 | |
| EU ×Sex |  | 0.033 | 0.037 | [-0.033, 0.097] | 0.370 | | 0.023 | 0.026 | [-0.023, 0.079] | 0.372 | |
| EU | CON_FPN2 | -0.004 | 0.014 | [-0.033, 0.022] | 0.778 | | -0.004 | 0.014 | [-0.033, 0.022] | 0.777 | |
| Threat |  | 0.009 | 0.007 | [-0.004, 0.024] | 0.199 | | 0.015 | 0.011 | [-0.007, 0.037] | 0.189 | |
| Age2 |  | -0.004 | 0.002 | [-0.007, -0.001] | 0.006 | | -0.035 | 0.013 | [-0.058, -0.009] | 0.006 | |
| Sex |  | -0.133 | 0.018 | [-0.168, -0.096] | < 0.001 | | -0.066 | 0.009 | [-0.084, -0.048] | < 0.001 | |
| Framewise Displacement |  | 0.365 | 0.108 | [0.162, 0.571] | 0.001 | | 0.055 | 0.015 | [0.026, 0.083] | < 0.001 | |
| Scanner Type |  | 0.089 | 0.027 | [0.030, 0.136] | 0.001 | | 0.059 | 0.018 | [0.022, 0.092] | 0.001 | |
| Race |  | 0.017 | 0.009 | [-0.001, 0.035] | 0.059 | | 0.023 | 0.012 | [-0.002, 0.045] | 0.052 | |
| CON_FPN0 |  | 9.150 | 0.339 | [8.448, 9.761] | < 0.001 | | 0.407 | 0.013 | [0.380, 0.430] | < 0.001 | |
| EU ×Sex |  | 0.001 | 0.028 | [-0.060, 0.053] | 0.971 | | 0.001 | 0.020 | [-0.041, 0.038] | 0.971 | |
| EU | DMN_FPN2 | 0.000 | 0.020 | [-0.041, 0.038] | 0.982 | | 0.000 | 0.020 | [-0.040, 0.039] | 0.982 | |
| Threat |  | -0.003 | 0.007 | [-0.017, 0.011] | 0.712 | | -0.004 | 0.012 | [-0.028, 0.018] | 0.714 | |
| Age2 |  | 0.004 | 0.001 | [0.002, 0.006] | < 0.001 | | 0.030 | 0.008 | [0.014, 0.045] | < 0.001 | |
| Sex |  | 0.030 | 0.016 | [0.001, 0.063] | 0.060 | | 0.015 | 0.008 | [0.001, 0.032] | 0.060 | |
| Framewise Displacement |  | 0.620 | 0.086 | [0.467, 0.803] | < 0.001 | | 0.093 | 0.011 | [0.072, 0.112] | < 0.001 | |
| Scanner Type |  | 0.078 | 0.043 | [0.000, 0.164] | 0.071 | | 0.052 | 0.025 | [0.003, 0.098] | 0.039 | |
| Race |  | 0.033 | 0.012 | [0.013, 0.059] | 0.004 | | 0.044 | 0.015 | [0.018, 0.077] | 0.003 | |
| DMN_FPN0 |  | 10.592 | 0.381 | [9.930, 11.403] | < 0.001 | | 0.450 | 0.013 | [0.427, 0.476] | < 0.001 | |
| EU ×Sex |  | -0.009 | 0.023 | [-0.051, 0.038] | 0.710 | | -0.006 | 0.016 | [-0.036, 0.027] | 0.709 | |

Note. EU = Environmental Unpredictability, CON = average correlations within the Cingulo-Opercular Network, DMN = average correlations within the Default Mode Network, FPN = average correlations within the Fronto-Parietal Network, CON_DMN = average correlations between Cingulo-Opercular Network and Default Mode Network, CON_FPN = average correlations between Cingulo-Opercular Network and Fronto-Parietal Network, DMN_FPN = average correlations between Default Mode Network and Fronto-Parietal Network. And the number following the abbreviation of the variable name represents the time of data collection, 0 = baseline, 2 = 2-year follow-up. Due to resampling in the cluster-robust standard error model, model fit indices (e.g., chi-square, CFI, and TLI) were not available with replicate weights. Based on the available indices (RMSEA = 0.000, SRMR = 0.000), the models fitted well. The reported *p* values were uncorrected.

Table S9. Three-way Interaction of Environmental Unpredictability × Sex ×Neighborhood Educational Deprivation in Predicting rsFC.

| Independent Variable and Covariates | Dependent Variable | Unstandardized | | | | Standardized | | | | |  |
| --- | --- | --- | --- | --- | --- | --- | --- | --- | --- | --- | --- |
|  |  | Coefficient | *SE* | *95% CI* | *p* | | Coefficient | *SE* | *95% CI* | *p* | |
| EU | CON2 | -0.020 | 0.014 | [-0.046, 0.009] | 0.151 | | -0.020 | 0.014 | [-0.046, 0.009] | 0.155 | |
| Threat |  | -0.011 | 0.008 | [-0.027, 0.003] | 0.139 | | -0.018 | 0.012 | [-0.043, 0.005] | 0.139 | |
| Age2 |  | 0.003 | 0.002 | [0.000, 0.006] | 0.061 | | 0.024 | 0.013 | [-0.001, 0.051] | 0.061 | |
| Sex |  | 0.074 | 0.032 | [0.015, 0.142] | 0.021 | | 0.037 | 0.016 | [0.007, 0.071] | 0.023 | |
| Framewise Displacement |  | -0.608 | 0.088 | [-0.748, -0.405] | < 0.001 | | -0.091 | 0.015 | [-0.116, -0.059] | < 0.001 | |
| Scanner Type |  | -0.033 | 0.043 | [-0.149, 0.027] | 0.446 | | -0.022 | 0.024 | [-0.076, 0.019] | 0.354 | |
| Race |  | -0.023 | 0.010 | [-0.042, -0.004] | 0.022 | | -0.030 | 0.013 | [-0.055, -0.006] | 0.017 | |
| CON0 |  | 7.255 | 0.294 | [6.677, 7.729] | < 0.001 | | 0.508 | 0.021 | [0.467, 0.542] | < 0.001 | |
| ND_E |  | 0.020 | 0.013 | [-0.006, 0.046] | 0.142 | | 0.020 | 0.014 | [-0.007, 0.046] | 0.146 | |
| EU × Sex × ND_E |  | -0.007 | 0.019 | [-0.049, 0.025] | 0.707 | | -0.005 | 0.013 | [-0.033, 0.019] | 0.705 | |
| EU | DMN2 | -0.034 | 0.010 | [-0.058, -0.018] | 0.001 | | -0.034 | 0.010 | [-0.056, -0.018] | < 0.001 | |
| Threat |  | -0.006 | 0.005 | [-0.016, 0.005] | 0.287 | | -0.009 | 0.009 | [-0.026, 0.008] | 0.290 | |
| Age2 |  | 0.002 | 0.002 | [-0.002, 0.007] | 0.359 | | 0.016 | 0.018 | [-0.018, 0.053] | 0.360 | |
| Sex |  | 0.165 | 0.022 | [0.123, 0.209] | < 0.001 | | 0.082 | 0.011 | [0.061, 0.104] | < 0.001 | |
| Framewise Displacement |  | -0.559 | 0.094 | [-0.752, -0.388] | < 0.001 | | -0.084 | 0.011 | [-0.104, -0.060] | < 0.001 | |
| Scanner Type |  | -0.145 | 0.095 | [-0.432, -0.029] | 0.129 | | -0.096 | 0.043 | [-0.201, -0.025] | 0.025 | |
| Race |  | -0.027 | 0.013 | [-0.056, -0.007] | 0.037 | | -0.035 | 0.017 | [-0.074, -0.009] | 0.035 | |
| DMN0 |  | 8.657 | 0.289 | [8.092, 9.131] | < 0.001 | | 0.498 | 0.016 | [0.467, 0.525] | < 0.001 | |
| ND_E |  | 0.036 | 0.020 | [0.003, 0.083] | 0.072 | | 0.036 | 0.020 | [0.003, 0.083] | 0.075 | |
| EU × Sex × ND_E |  | 0.037 | 0.019 | [0.000, 0.074] | 0.052 | | 0.026 | 0.013 | [0.000, 0.052] | 0.051 | |
| EU | FPN2 | 0.008 | 0.010 | [-0.015, 0.027] | 0.439 | | 0.008 | 0.010 | [-0.014, 0.027] | 0.440 | |
| Threat |  | -0.009 | 0.006 | [-0.020, 0.002] | 0.119 | | -0.014 | 0.009 | [-0.032, 0.004] | 0.121 | |
| Age2 |  | 0.001 | 0.002 | [-0.002, 0.005] | 0.392 | | 0.011 | 0.013 | [-0.013, 0.037] | 0.393 | |
| Sex |  | 0.122 | 0.026 | [0.071, 0.173] | < 0.001 | | 0.061 | 0.013 | [0.035, 0.086] | < 0.001 | |
| Framewise Displacement |  | -0.517 | 0.115 | [-0.724, -0.295] | < 0.001 | | -0.077 | 0.015 | [-0.104, -0.048] | < 0.001 | |
| Scanner Type |  | -0.031 | 0.073 | [-0.253, 0.054] | 0.666 | | -0.021 | 0.038 | [-0.113, 0.041] | 0.587 | |
| Race |  | -0.013 | 0.013 | [-0.037, 0.011] | 0.310 | | -0.017 | 0.017 | [-0.051, 0.014] | 0.316 | |
| FPN0 |  | 9.228 | 0.302 | [8.687, 9.797] | < 0.001 | | 0.518 | 0.013 | [0.494, 0.543] | < 0.001 | |
| ND_E |  | -0.012 | 0.023 | [-0.053, 0.038] | 0.593 | | -0.012 | 0.023 | [-0.053, 0.038] | 0.592 | |
| EU × Sex × ND_E |  | 0.019 | 0.021 | [-0.020, 0.061] | 0.362 | | 0.013 | 0.015 | [-0.013, 0.043] | 0.363 | |
| EU | CON_DMN2 | 0.029 | 0.008 | [0.013, 0.046] | 0.001 | | 0.029 | 0.008 | [0.013, 0.046] | 0.001 | |
| Threat |  | -0.003 | 0.005 | [-0.010, 0.008] | 0.537 | | -0.005 | 0.007 | [-0.017, 0.013] | 0.540 | |
| Age2 |  | -0.006 | 0.002 | [-0.010, -0.002] | 0.001 | | -0.049 | 0.015 | [-0.079, -0.019] | 0.001 | |
| Sex |  | -0.135 | 0.031 | [-0.199, -0.076] | < 0.001 | | -0.067 | 0.016 | [-0.099, -0.038] | < 0.001 | |
| Framewise Displacement |  | 1.042 | 0.151 | [0.748, 1.326] | < 0.001 | | 0.156 | 0.015 | [0.126, 0.185] | < 0.001 | |
| Scanner Type |  | 0.138 | 0.076 | [0.033, 0.346] | 0.071 | | 0.091 | 0.035 | [0.029, 0.170] | 0.010 | |
| Race |  | 0.024 | 0.013 | [-0.001, 0.048] | 0.062 | | 0.032 | 0.017 | [-0.001, 0.061] | 0.056 | |
| CON_DMN0 |  | 7.619 | 0.238 | [7.217, 8.181] | < 0.001 | | 0.408 | 0.014 | [0.383, 0.439] | < 0.001 | |
| ND_E |  | -0.030 | 0.016 | [-0.063, -0.002] | 0.053 | | -0.030 | 0.015 | [-0.062, -0.002] | 0.050 | |
| EU × Sex × ND_E |  | -0.026 | 0.019 | [-0.061, 0.013] | 0.173 | | -0.018 | 0.014 | [-0.044, 0.009] | 0.178 | |
| EU | CON_FPN2 | -0.001 | 0.010 | [-0.021, 0.020] | 0.910 | | -0.001 | 0.010 | [-0.020, 0.021] | 0.909 | |
| Threat |  | 0.009 | 0.007 | [-0.004, 0.024] | 0.203 | | 0.015 | 0.011 | [-0.007, 0.037] | 0.192 | |
| Age2 |  | -0.004 | 0.002 | [-0.007, -0.001] | 0.006 | | -0.035 | 0.013 | [-0.058, -0.010] | 0.005 | |
| Sex |  | -0.130 | 0.019 | [-0.167, -0.093] | < 0.001 | | -0.065 | 0.009 | [-0.084, -0.046] | < 0.001 | |
| Framewise Displacement |  | 0.367 | 0.108 | [0.165, 0.575] | 0.001 | | 0.055 | 0.014 | [0.027, 0.083] | < 0.001 | |
| Scanner Type |  | 0.089 | 0.027 | [0.029, 0.137] | 0.001 | | 0.059 | 0.018 | [0.022, 0.092] | 0.001 | |
| Race |  | 0.017 | 0.009 | [-0.001, 0.035] | 0.067 | | 0.022 | 0.012 | [-0.002, 0.045] | 0.060 | |
| CON_FPN0 |  | 9.149 | 0.339 | [8.447, 9.763] | < 0.001 | | 0.407 | 0.013 | [0.380, 0.430] | < 0.001 | |
| ND_E |  | -0.001 | 0.014 | [-0.029, 0.027] | 0.939 | | -0.001 | 0.014 | [-0.028, 0.027] | 0.939 | |
| EU × Sex × ND_E |  | 0.022 | 0.016 | [-0.010, 0.051] | 0.164 | | 0.015 | 0.011 | [-0.008, 0.035] | 0.156 | |
| EU | DMN_FPN2 | -0.008 | 0.013 | [-0.035, 0.017] | 0.553 | | -0.008 | 0.013 | [-0.033, 0.018] | 0.547 | |
| Threat |  | -0.003 | 0.007 | [-0.017, 0.011] | 0.687 | | -0.005 | 0.012 | [-0.028, 0.018] | 0.689 | |
| Age2 |  | 0.004 | 0.001 | [0.002, 0.006] | < 0.001 | | 0.030 | 0.008 | [0.014, 0.045] | < 0.001 | |
| Sex |  | 0.029 | 0.016 | [0.000, 0.064] | 0.076 | | 0.014 | 0.008 | [0.000, 0.032] | 0.076 | |
| Framewise Displacement |  | 0.614 | 0.084 | [0.463, 0.791] | < 0.001 | | 0.092 | 0.010 | [0.071, 0.111] | < 0.001 | |
| Scanner Type |  | 0.078 | 0.042 | [0.002, 0.162] | 0.060 | | 0.052 | 0.024 | [0.005, 0.097] | 0.032 | |
| Race |  | 0.030 | 0.012 | [0.010, 0.058] | 0.011 | | 0.040 | 0.015 | [0.014, 0.073] | 0.008 | |
| DMN_FPN0 |  | 10.586 | 0.383 | [9.921, 11.409] | < 0.001 | | 0.450 | 0.013 | [0.427, 0.476] | < 0.001 | |
| ND_E |  | -0.023 | 0.017 | [-0.065, 0.004] | 0.171 | | -0.023 | 0.017 | [-0.063, 0.003] | 0.159 | |
| EU × Sex × ND_E |  | 0.000 | 0.012 | [-0.023, 0.026] | 0.986 | | 0.000 | 0.009 | [-0.016, 0.018] | 0.986 | |

Note. EU = Environmental Unpredictability, ND_E = Neighborhood Deprivation in Education domain, CON = average correlations within the Cingulo-Opercular Network, DMN = average correlations within the Default Mode Network, FPN = average correlations within the Fronto-Parietal Network, CON_DMN = average correlations between Cingulo-Opercular Network and Default Mode Network, CON_FPN = average correlations between Cingulo-Opercular Network and Fronto-Parietal Network, DMN_FPN = average correlations between Default Mode Network and Fronto-Parietal Network. And the number following the abbreviation of the variable name represents the time of data collection, 0 = baseline, 2 = 2-year follow-up. Due to resampling in the cluster-robust standard error model, model fit indices (e.g., chi-square, CFI, and TLI) were not available with replicate weights. Based on the available indices (RMSEA = 0.000, SRMR = 0.000), the models fitted well. The reported *p* values were uncorrected.

Table S10. Three-way Interaction of Environmental Unpredictability × Sex ×Neighborhood Health Deprivation in Predicting rsFC.

| Independent Variable and Covariates | Dependent Variable | Unstandardized | | | | Standardized | | | | |  |
| --- | --- | --- | --- | --- | --- | --- | --- | --- | --- | --- | --- |
|  |  | Coefficient | *SE* | *95% CI* | *p* | | Coefficient | *SE* | *95% CI* | *p* | |
| EU | CON2 | -0.016 | 0.013 | [-0.040, 0.010] | 0.219 | | -0.016 | 0.013 | [-0.041, 0.010] | 0.224 | |
| Threat |  | -0.011 | 0.008 | [-0.027, 0.004] | 0.175 | | -0.017 | 0.012 | [-0.042, 0.007] | 0.176 | |
| Age2 |  | 0.003 | 0.002 | [0.000, 0.006] | 0.073 | | 0.022 | 0.012 | [-0.002, 0.047] | 0.074 | |
| Sex |  | 0.079 | 0.031 | [0.022, 0.143] | 0.010 | | 0.039 | 0.016 | [0.011, 0.072] | 0.012 | |
| Framewise Displacement |  | -0.588 | 0.088 | [-0.730, -0.384] | < 0.001 | | -0.088 | 0.015 | [-0.113, -0.055] | < 0.001 | |
| Scanner Type |  | -0.036 | 0.040 | [-0.141, 0.017] | 0.375 | | -0.024 | 0.021 | [-0.074, 0.011] | 0.270 | |
| Race |  | -0.018 | 0.009 | [-0.038, -0.001] | 0.050 | | -0.024 | 0.012 | [-0.048, -0.002] | 0.040 | |
| CON0 |  | 7.156 | 0.288 | [6.589, 7.646] | < 0.001 | | 0.501 | 0.021 | [0.461, 0.535] | < 0.001 | |
| ND_HE |  | 0.057 | 0.014 | [0.032, 0.086] | < 0.001 | | 0.057 | 0.015 | [0.030, 0.087] | < 0.001 | |
| EU × Sex × ND_HE |  | 0.003 | 0.020 | [-0.039, 0.039] | 0.892 | | 0.002 | 0.015 | [-0.031, 0.028] | 0.891 | |
| EU | DMN2 | -0.033 | 0.010 | [-0.056, -0.017] | 0.001 | | -0.033 | 0.010 | [-0.055, -0.017] | 0.001 | |
| Threat |  | -0.005 | 0.005 | [-0.015, 0.006] | 0.360 | | -0.008 | 0.008 | [-0.024, 0.009] | 0.362 | |
| Age2 |  | 0.002 | 0.002 | [-0.002, 0.006] | 0.436 | | 0.013 | 0.017 | [-0.019, 0.048] | 0.438 | |
| Sex |  | 0.166 | 0.021 | [0.126, 0.208] | < 0.001 | | 0.083 | 0.010 | [0.062, 0.103] | < 0.001 | |
| Framewise Displacement |  | -0.535 | 0.094 | [-0.725, -0.365] | < 0.001 | | -0.080 | 0.011 | [-0.100, -0.056] | < 0.001 | |
| Scanner Type |  | -0.146 | 0.091 | [-0.421, -0.037] | 0.108 | | -0.097 | 0.040 | [-0.193, -0.030] | 0.015 | |
| Race |  | -0.022 | 0.012 | [-0.050, -0.003] | 0.070 | | -0.029 | 0.016 | [-0.065, -0.004] | 0.066 | |
| DMN0 |  | 8.594 | 0.287 | [8.034, 9.082] | < 0.001 | | 0.494 | 0.016 | [0.464, 0.523] | < 0.001 | |
| ND_HE |  | 0.071 | 0.027 | [0.029, 0.124] | 0.008 | | 0.071 | 0.029 | [0.028, 0.139] | 0.014 | |
| EU × Sex × ND_HE |  | 0.026 | 0.017 | [-0.009, 0.058] | 0.120 | | 0.020 | 0.012 | [-0.007, 0.041] | 0.103 | |
| EU | FPN2 | 0.010 | 0.011 | [-0.013, 0.030] | 0.363 | | 0.010 | 0.011 | [-0.013, 0.030] | 0.365 | |
| Threat |  | -0.008 | 0.006 | [-0.020, 0.003] | 0.135 | | -0.013 | 0.009 | [-0.032, 0.004] | 0.136 | |
| Age2 |  | 0.001 | 0.002 | [-0.002, 0.005] | 0.405 | | 0.011 | 0.013 | [-0.013, 0.037] | 0.406 | |
| Sex |  | 0.122 | 0.027 | [0.069, 0.172] | < 0.001 | | 0.061 | 0.013 | [0.034, 0.086] | < 0.001 | |
| Framewise Displacement |  | -0.508 | 0.116 | [-0.714, -0.283] | < 0.001 | | -0.076 | 0.015 | [-0.103, -0.046] | < 0.001 | |
| Scanner Type |  | -0.031 | 0.072 | [-0.249, 0.054] | 0.665 | | -0.021 | 0.038 | [-0.111, 0.042] | 0.583 | |
| Race |  | -0.010 | 0.013 | [-0.033, 0.015] | 0.435 | | -0.013 | 0.017 | [-0.046, 0.018] | 0.438 | |
| FPN0 |  | 9.216 | 0.305 | [8.672, 9.791] | < 0.001 | | 0.517 | 0.013 | [0.494, 0.542] | < 0.001 | |
| ND_HE |  | 0.012 | 0.025 | [-0.030, 0.066] | 0.643 | | 0.012 | 0.025 | [-0.028, 0.069] | 0.641 | |
| EU × Sex × ND_HE |  | 0.015 | 0.022 | [-0.032, 0.054] | 0.501 | | 0.011 | 0.016 | [-0.022, 0.040] | 0.495 | |
| EU | CON_DMN2 | 0.026 | 0.009 | [0.010, 0.043] | 0.003 | | 0.026 | 0.009 | [0.010, 0.043] | 0.004 | |
| Threat |  | -0.004 | 0.005 | [-0.012, 0.007] | 0.407 | | -0.006 | 0.008 | [-0.020, 0.012] | 0.411 | |
| Age2 |  | -0.006 | 0.002 | [-0.009, -0.002] | 0.001 | | -0.046 | 0.014 | [-0.074, -0.018] | 0.001 | |
| Sex |  | -0.136 | 0.031 | [-0.197, -0.076] | < 0.001 | | -0.068 | 0.015 | [-0.098, -0.038] | < 0.001 | |
| Framewise Displacement |  | 1.008 | 0.150 | [0.718, 1.295] | < 0.001 | | 0.151 | 0.015 | [0.119, 0.179] | < 0.001 | |
| Scanner Type |  | 0.138 | 0.072 | [0.038, 0.323] | 0.056 | | 0.091 | 0.033 | [0.031, 0.161] | 0.006 | |
| Race |  | 0.017 | 0.012 | [-0.006, 0.039] | 0.142 | | 0.023 | 0.015 | [-0.007, 0.050] | 0.135 | |
| CON_DMN0 |  | 7.6013 | 0.239 | [7.217, 8.165] | < 0.001 | | 0.407 | 0.014 | [0.384, 0.438] | < 0.001 | |
| ND_HE |  | -0.081 | 0.021 | [-0.126, -0.043] | < 0.001 | | -0.081 | 0.022 | [-0.128, -0.042] | < 0.001 | |
| EU × Sex × ND_HE |  | -0.017 | 0.020 | [-0.053, 0.026] | 0.390 | | -0.013 | 0.015 | [-0.041, 0.019] | 0.394 | |
| EU | CON_FPN2 | -0.007 | 0.010 | [-0.025, 0.016] | 0.518 | | -0.007 | 0.010 | [-0.024, 0.017] | 0.511 | |
| Threat |  | 0.009 | 0.007 | [-0.005, 0.024] | 0.234 | | 0.014 | 0.011 | [-0.008, 0.037] | 0.224 | |
| Age2 |  | -0.004 | 0.002 | [-0.007, -0.001] | 0.007 | | -0.034 | 0.012 | [-0.056, -0.008] | 0.006 | |
| Sex |  | -0.135 | 0.019 | [-0.172, -0.098] | < 0.001 | | -0.068 | 0.009 | [-0.086, -0.050] | < 0.001 | |
| Framewise Displacement |  | 0.350 | 0.105 | [0.156, 0.555] | 0.001 | | 0.052 | 0.014 | [0.025, 0.080] | < 0.001 | |
| Scanner Type |  | 0.089 | 0.030 | [0.021, 0.141] | 0.003 | | 0.059 | 0.021 | [0.016, 0.097] | 0.004 | |
| Race |  | 0.014 | 0.009 | [-0.005, 0.031] | 0.146 | | 0.018 | 0.012 | [-0.007, 0.041] | 0.138 | |
| CON_FPN0 |  | 9.142 | 0.345 | [8.445, 9.773] | < 0.001 | | 0.407 | 0.013 | [0.380, 0.431] | < 0.001 | |
| ND_HE |  | -0.027 | 0.022 | [-0.072, 0.015] | 0.213 | | -0.027 | 0.022 | [-0.074, 0.013] | 0.216 | |
| EU × Sex × ND_HE |  | -0.007 | 0.015 | [-0.037, 0.019] | 0.617 | | -0.005 | 0.011 | [-0.029, 0.014] | 0.617 | |
| EU | DMN_FPN2 | -0.006 | 0.014 | [-0.034, 0.020] | 0.659 | | -0.006 | 0.013 | [-0.033, 0.020] | 0.654 | |
| Threat |  | -0.003 | 0.007 | [-0.017, 0.011] | 0.651 | | -0.005 | 0.012 | [-0.029, 0.017] | 0.654 | |
| Age2 |  | 0.004 | 0.001 | [0.002, 0.006] | < 0.001 | | 0.031 | 0.008 | [0.015, 0.046] | < 0.001 | |
| Sex |  | 0.030 | 0.016 | [0.001, 0.065] | 0.065 | | 0.015 | 0.008 | [0.000, 0.032] | 0.065 | |
| Framewise Displacement |  | 0.608 | 0.085 | [0.456, 0.787] | < 0.001 | | 0.091 | 0.010 | [0.070, 0.111] | < 0.001 | |
| Scanner Type |  | 0.078 | 0.040 | [0.003, 0.159] | 0.053 | | 0.052 | 0.024 | [0.006, 0.096] | 0.028 | |
| Race |  | 0.029 | 0.012 | [0.009, 0.058] | 0.017 | | 0.039 | 0.016 | [0.012, 0.074] | 0.012 | |
| DMN_FPN0 |  | 10.598 | 0.386 | [9.919, 11.429] | < 0.001 | | 0.451 | 0.013 | [0.427, 0.476] | < 0.001 | |
| ND_HE |  | -0.026 | 0.021 | [-0.073, 0.009] | 0.219 | | -0.026 | 0.021 | [-0.075, 0.008] | 0.226 | |
| EU × Sex × ND_HE |  | 0.016 | 0.020 | [-0.022, 0.055] | 0.414 | | 0.012 | 0.015 | [-0.016, 0.041] | 0.411 | |

Note. EU = Environmental Unpredictability, ND_HE = Neighborhood Deprivation in Health and Environment domain, CON = average correlations within the Cingulo-Opercular Network, DMN = average correlations within the Default Mode Network, FPN = average correlations within the Fronto-Parietal Network, CON_DMN = average correlations between Cingulo-Opercular Network and Default Mode Network, CON_FPN = average correlations between Cingulo-Opercular Network and Fronto-Parietal Network, DMN_FPN = average correlations between Default Mode Network and Fronto-Parietal Network. And the number following the abbreviation of the variable name represents the time of data collection, 0 = baseline, 2 = 2-year follow-up. Due to resampling in the cluster-robust standard error model, model fit indices (e.g., chi-square, CFI, and TLI) were not available with replicate weights. Based on the available indices (RMSEA = 0.000, SRMR = 0.000), the models fitted well. The reported *p* values were uncorrected.

Table S11. Three-way Interaction of Environmental Unpredictability × Sex ×Neighborhood Socioeconomic Deprivation in Predicting rsFC.

| Independent Variable and Covariates | Dependent Variable | Unstandardized | | | | Standardized | | | | |  |
| --- | --- | --- | --- | --- | --- | --- | --- | --- | --- | --- | --- |
|  |  | Coefficient | *SE* | *95% CI* | *p* | | Coefficient | *SE* | *95% CI* | *p* | |
| EU | CON2 | -0.015 | 0.014 | [-0.041, 0.014] | 0.284 | | -0.015 | 0.014 | [-0.042, 0.014] | 0.288 | |
| Threat |  | -0.010 | 0.008 | [-0.026, 0.004] | 0.175 | | -0.017 | 0.012 | [-0.041, 0.007] | 0.0176 | |
| Age2 |  | 0.003 | 0.002 | [0.000, 0.006] | 0.070 | | 0.023 | 0.013 | [-0.001, 0.048] | 0.070 | |
| Sex |  | 0.078 | 0.030 | [0.022, 0.142] | 0.010 | | 0.039 | 0.015 | [0.011, 0.072] | 0.011 | |
| Framewise Displacement |  | -0.599 | 0.087 | [-0.739, -0.396] | < 0.001 | | -0.089 | 0.015 | [-0.115, -0.057] | < 0.001 | |
| Scanner Type |  | -0.033 | 0.043 | [-0.152, 0.025] | 0.445 | | -0.022 | 0.023 | [-0.077, 0.017] | 0.347 | |
| Race |  | -0.018 | 0.009 | [-0.036, -0.002] | 0.039 | | -0.024 | 0.011 | [-0.046, -0.003] | 0.032 | |
| CON0 |  | 7.176 | 0.276 | [6.612, 7.619] | < 0.001 | | 0.503 | 0.020 | [0.464, 0.536] | < 0.001 | |
| ND_SE |  | 0.046 | 0.015 | [0.017, 0.073] | 0.002 | | 0.046 | 0.015 | [0.017, 0.074] | 0.002 | |
| EU × Sex × ND_SE |  | 0.012 | 0.017 | [-0.021, 0.047] | 0.494 | | 0.009 | 0.013 | [-0.016, 0.033] | 0.488 | |
| EU | DMN2 | -0.035 | 0.010 | [-0.058, -0.019] | < 0.001 | | -0.035 | 0.009 | [-0.056, -0.020] | < 0.001 | |
| Threat |  | -0.005 | 0.005 | [-0.015, 0.006] | 0.374 | | -0.008 | 0.009 | [-0.024, 0.009] | 0.376 | |
| Age2 |  | 0.002 | 0.002 | [-0.002, 0.006] | 0.410 | | 0.014 | 0.018 | [-0.019, 0.050] | 0.411 | |
| Sex |  | 0.164 | 0.021 | [0.123, 0.206] | < 0.001 | | 0.082 | 0.011 | [0.061, 0.103] | < 0.001 | |
| Framewise Displacement |  | -0.554 | 0.094 | [-0.744, -0.383] | < 0.001 | | -0.083 | 0.011 | [-0.103, -0.059] | < 0.001 | |
| Scanner Type |  | -0.143 | 0.097 | [-0.438, -0.028] | 0.138 | | -0.095 | 0.043 | [-0.204, -0.025] | 0.029 | |
| Race |  | -0.023 | 0.012 | [-0.052, -0.004] | 0.062 | | -0.031 | 0.017 | [-0.068, -0.005] | 0.062 | |
| DMN0 |  | 8.626 | 0.284 | [8.081, 9.100] | < 0.001 | | 0.496 | 0.016 | [0.466, 0.524] | < 0.001 | |
| ND_SE |  | 0.047 | 0.018 | [0.012, 0.082] | 0.009 | | 0.047 | 0.017 | [0.012, 0.078] | 0.007 | |
| EU × Sex × ND_SE |  | 0.027 | 0.018 | [-0.007, 0.064] | 0.142 | | 0.020 | 0.013 | [-0.005, 0.046] | 0.134 | |
| EU | FPN2 | 0.007 | 0.010 | [-0.016, 0.025] | 0.494 | | 0.007 | 0.010 | [-0.016, 0.025] | 0.494 | |
| Threat |  | -0.009 | 0.005 | [-0.020, 0.002] | 0.111 | | -0.014 | 0.009 | [-0.032, 0.003] | 0.113 | |
| Age2 |  | 0.001 | 0.002 | [-0.002, 0.005] | 0.371 | | 0.011 | 0.013 | [-0.013, 0.037] | 0.372 | |
| Sex |  | 0.121 | 0.026 | [0.071, 0.171] | < 0.001 | | 0.061 | 0.013 | [0.035, 0.086] | < 0.001 | |
| Framewise Displacement |  | -0.520 | 0.115 | [-0.729, -0.298] | < 0.001 | | -0.078 | 0.015 | [-0.105, -0.048] | < 0.001 | |
| Scanner Type |  | -0.032 | 0.072 | [-0.252, 0.053] | 0.658 | | -0.021 | 0.038 | [-0.112, 0.040] | 0.576 | |
| Race |  | -0.013 | 0.012 | [-0.038, 0.009] | 0.284 | | -0.018 | 0.017 | [-0.053, 0.012] | 0.291 | |
| FPN0 |  | 9.219 | 0.304 | [8.674, 9.785] | < 0.001 | | 0.517 | 0.013 | [0.493, 0.542] | < 0.001 | |
| ND_SE |  | -0.013 | 0.017 | [-0.047, 0.022] | 0.456 | | -0.013 | 0.017 | [-0.048, 0.021] | 0.455 | |
| EU × Sex × ND_SE |  | 0.015 | 0.017 | [-0.020, 0.049] | 0.384 | | 0.011 | 0.013 | [-0.013, 0.037] | 0.390 | |
| EU | CON_DMN2 | 0.026 | 0.008 | [0.010, 0.042] | 0.002 | | 0.026 | 0.009 | [0.010, 0.042] | 0.002 | |
| Threat |  | -0.004 | 0.005 | [-0.012, 0.007] | 0.373 | | -0.007 | 0.008 | [-0.020, 0.011] | 0.378 | |
| Age2 |  | -0.006 | 0.002 | [-0.010, -0.002] | 0.001 | | -0.047 | 0.015 | [-0.077, -0.018] | 0.001 | |
| Sex |  | -0.136 | 0.031 | [-0.197, -0.077] | < 0.001 | | -0.068 | 0.015 | [-0.099, -0.038] | < 0.001 | |
| Framewise Displacement |  | 1.030 | 0.149 | [0.736, 1.310] | < 0.001 | | 0.154 | 0.015 | [0.123, 0.182] | < 0.001 | |
| Scanner Type |  | 0.135 | 0.077 | [0.032, 0.349] | 0.077 | | 0.089 | 0.035 | [0.027, 0.168] | 0.011 | |
| Race |  | 0.018 | 0.012 | [-0.006, 0.039] | 0.131 | | 0.024 | 0.016 | [-0.008, 0.051] | 0.127 | |
| CON_DMN0 |  | 7.581 | 0.233 | [7.187, 8.130] | < 0.001 | | 0.406 | 0.013 | [0.382, 0.436] | < 0.001 | |
| ND_SE |  | -0.059 | 0.013 | [-0.084, -0.034] | < 0.001 | | -0.059 | 0.014 | [-0.087, -0.033] | < 0.001 | |
| EU × Sex × ND_SE |  | -0.022 | 0.018 | [-0.054, 0.016] | 0.218 | | -0.017 | 0.014 | [-0.041, 0.012] | 0.222 | |
| EU | CON_FPN2 | -0.006 | 0.010 | [-0.025, 0.015] | 0.546 | | -0.006 | 0.010 | [-0.024, 0.016] | 0.539 | |
| Threat |  | 0.009 | 0.007 | [-0.005, 0.023] | 0.228 | | 0.014 | 0.011 | [-0.008, 0.036] | 0.217 | |
| Age2 |  | -0.004 | 0.002 | [-0.007, -0.001] | 0.006 | | -0.034 | 0.012 | [-0.057, -0.009] | 0.006 | |
| Sex |  | -0.135 | 0.018 | [-0.171, -0.099] | < 0.001 | | -0.067 | 0.009 | [-0.085, -0.050] | < 0.001 | |
| Framewise Displacement |  | 0.359 | 0.108 | [0.159, 0.568] | 0.001 | | 0.054 | 0.014 | [0.025, 0.082] | < 0.001 | |
| Scanner Type |  | 0.089 | 0.027 | [0.027, 0.135] | 0.001 | | 0.059 | 0.018 | [0.021, 0.092] | 0.001 | |
| Race |  | 0.015 | 0.010 | [-0.004, 0.033] | 0.129 | | 0.019 | 0.012 | [-0.006, 0.043] | 0.120 | |
| CON_FPN0 |  | 9.140 | 0.338 | [8.442, 9.754] | < 0.001 | | 0.407 | 0.013 | [0.380, 0.430] | < 0.001 | |
| ND_SE |  | -0.017 | 0.015 | [-0.045, 0.014] | 0.262 | | -0.017 | 0.015 | [-0.047, 0.013] | 0.267 | |
| EU × Sex × ND_SE |  | -0.006 | 0.015 | [-0.035, 0.024] | 0.673 | | -0.005 | 0.011 | [-0.025, 0.018] | 0.670 | |
| EU | DMN_FPN2 | -0.006 | 0.014 | [-0.034, 0.021] | 0.652 | | -0.006 | 0.014 | [-0.032, 0.022] | 0.647 | |
| Threat |  | -0.003 | 0.007 | [-0.017, 0.011] | 0.649 | | -0.005 | 0.011 | [-0.028, 0.017] | 0.652 | |
| Age2 |  | 0.004 | 0.001 | [0.002, 0.006] | < 0.001 | | 0.030 | 0.008 | [0.015, 0.045] | < 0.001 | |
| Sex |  | 0.034 | 0.017 | [0.003, 0.071] | 0.047 | | 0.017 | 0.008 | [0.002, 0.035] | 0.047 | |
| Framewise Displacement |  | 0.610 | 0.084 | [0.460, 0.789] | < 0.001 | | 0.091 | 0.011 | [0.070, 0.111] | < 0.001 | |
| Scanner Type |  | 0.077 | 0.042 | [0.002, 0.164] | 0.071 | | 0.051 | 0.024 | [0.004, 0.097] | 0.038 | |
| Race |  | 0.027 | 0.012 | [0.007, 0.054] | 0.018 | | 0.036 | 0.015 | [0.010, 0.069] | 0.014 | |
| DMN_FPN0 |  | 10.589 | 0.385 | [9.920, 11.418] | < 0.001 | | 0.450 | 0.013 | [0.426, 0.476] | < 0.001 | |
| ND_SE |  | -0.033 | 0.016 | [-0.067, -0.004] | 0.042 | | -0.033 | 0.016 | [-0.065, -0.005] | 0.035 | |
| EU × Sex × ND_SE |  | 0.033 | 0.016 | [0.005, 0.068] | 0.044 | | 0.024 | 0.013 | [0.003, 0.054] | 0.062 | |

Note. EU = Environmental Unpredictability, ND_SE = Neighborhood Deprivation in Social and Economic domain, CON = average correlations within the Cingulo-Opercular Network, DMN = average correlations within the Default Mode Network, FPN = average correlations within the Fronto-Parietal Network, CON_DMN = average correlations between Cingulo-Opercular Network and Default Mode Network, CON_FPN = average correlations between Cingulo-Opercular Network and Fronto-Parietal Network, DMN_FPN = average correlations between Default Mode Network and Fronto-Parietal Network. And the number following the abbreviation of the variable name represents the time of data collection, 0 = baseline, 2 = 2-year follow-up. Due to resampling in the cluster-robust standard error model, model fit indices (e.g., chi-square, CFI, and TLI) were not available with replicate weights. Based on the available indices (RMSEA = 0.000, SRMR = 0.000), the models fitted well. The reported *p* values were uncorrected.

Table S12. Three-way Interaction of Environmental Unpredictability × Sex × Neighborhood Deprivation in Predicting rsFC.

| Independent Variable and Covariates | Dependent Variable | Unstandardized | | | | Standardized | | | | |  |
| --- | --- | --- | --- | --- | --- | --- | --- | --- | --- | --- | --- |
|  |  | Coefficient | *SE* | *95% CI* | *p* | | Coefficient | *SE* | *95% CI* | *p* | |
| EU | CON2 | -0.015 | 0.014 | [-0.041, 0.014] | 0.285 | | -0.015 | 0.014 | [-0.042, 0.014] | 0.289 | |
| Threat |  | -0.011 | 0.008 | [-0.026, 0.004] | 0.169 | | -0.017 | 0.012 | [-0.042, 0.006] | 0.170 | |
| Age2 |  | 0.003 | 0.002 | [0.000, 0.006] | 0.065 | | 0.023 | 0.013 | [-0.001, 0.048] | 0.065 | |
| Sex |  | 0.078 | 0.031 | [0.021, 0.143] | 0.011 | | 0.039 | 0.016 | [0.010, 0.072] | 0.012 | |
| Framewise Displacement |  | -0.598 | 0.088 | [-0.739, -0.394] | < 0.001 | | -0.089 | 0.015 | [-0.115, -0.057] | < 0.001 | |
| Scanner Type |  | -0.033 | 0.042 | [-0.149, 0.023] | 0.430 | | -0.022 | 0.023 | [-0.076, 0.016] | 0.331 | |
| Race |  | -0.018 | 0.009 | [-0.037, -0.001] | 0.045 | | -0.024 | 0.012 | [-0.047, -0.002] | 0.037 | |
| CON0 |  | 7.179 | 0.280 | [6.611, 7.631] | < 0.001 | | 0.503 | 0.020 | [0.463, 0.537] | < 0.001 | |
| ND |  | 0.046 | 0.014 | [0.018, 0.071] | 0.001 | | 0.046 | 0.014 | [0.018, 0.072] | 0.001 | |
| EU × Sex × ND |  | 0.007 | 0.017 | [-0.027, 0.041] | 0.669 | | 0.005 | 0.013 | [-0.020, 0.030] | 0.667 | |
| EU | DMN2 | -0.033 | 0.010 | [-0.056, -0.017] | 0.001 | | -0.033 | 0.009 | [-0.054, -0.018] | < 0.001 | |
| Threat |  | -0.005 | 0.005 | [-0.015, 0.006] | 0.369 | | -0.008 | 0.009 | [-0.024, 0.009] | 0.370 | |
| Age2 |  | 0.002 | 0.002 | [-0.002, 0.006] | 0.402 | | 0.015 | 0.018 | [-0.019, 0.050] | 0.404 | |
| Sex |  | 0.166 | 0.022 | [0.124, 0.209] | < 0.001 | | 0.083 | 0.011 | [0.062, 0.104] | < 0.001 | |
| Framewise Displacement |  | -0.550 | 0.093 | [-0.739, -0.379] | < 0.001 | | -0.082 | 0.011 | [-0.102, -0.059] | < 0.001 | |
| Scanner Type |  | -0.144 | 0.095 | [-0.433, -0.030] | 0.131 | | -0.095 | 0.043 | [-0.202, -0.026] | 0.025 | |
| Race |  | -0.023 | 0.012 | [-0.051, -0.003] | 0.065 | | -0.030 | 0.016 | [-0.067, -0.005] | 0.064 | |
| DMN0 |  | 8.616 | 0.288 | [8.062, 9.097] | < 0.001 | | 0.495 | 0.016 | [0.465, 0.524] | < 0.001 | |
| ND |  | 0.052 | 0.020 | [0.017, 0.093] | 0.009 | | 0.052 | 0.020 | [0.017, 0.092] | 0.008 | |
| EU × Sex × ND |  | 0.032 | 0.019 | [-0.005, 0.068] | 0.094 | | 0.023 | 0.014 | [-0.004, 0.049] | 0.087 | |
| EU | FPN2 | 0.008 | 0.010 | [-0.015, 0.026] | 0.453 | | 0.008 | 0.010 | [-0.015, 0.026] | 0.453 | |
| Threat |  | -0.009 | 0.005 | [-0.020, 0.002] | 0.111 | | -0.014 | 0.009 | [-0.032, 0.003] | 0.113 | |
| Age2 |  | 0.001 | 0.002 | [-0.002, 0.005] | 0.379 | | 0.011 | 0.013 | [-0.013, 0.037] | 0.380 | |
| Sex |  | 0.122 | 0.026 | [0.071, 0.172] | < 0.001 | | 0.061 | 0.013 | [0.035, 0.086] | < 0.001 | |
| Framewise Displacement |  | -0.519 | 0.116 | [-0.728, -0.296] | < 0.001 | | -0.078 | 0.015 | [-0.104, -0.052] | < 0.001 | |
| Scanner Type |  | -0.032 | 0.072 | [-0.253, 0.053] | 0.662 | | -0.021 | 0.038 | [-0.112, 0.031] | 0.581 | |
| Race |  | -0.013 | 0.012 | [-0.037, 0.009] | 0.282 | | -0.017 | 0.016 | [-0.051, 0.007] | 0.290 | |
| FPN0 |  | 9.222 | 0.303 | [8.680, 9.790] | < 0.001 | | 0.518 | 0.013 | [0.494, 0.539] | < 0.001 | |
| ND |  | -0.011 | 0.020 | [-0.050, 0.028] | 0.587 | | -0.011 | 0.020 | [-0.049, 0.023] | 0.584 | |
| EU × Sex × ND |  | 0.017 | 0.018 | [-0.020, 0.053] | 0.347 | | 0.013 | 0.014 | [-0.014, 0.035] | 0.353 | |
| EU | CON_DMN2 | 0.025 | 0.008 | [0.009, 0.041] | 0.003 | | 0.025 | 0.009 | [0.009, 0.042] | 0.004 | |
| Threat |  | -0.004 | 0.005 | [-0.012, 0.007] | 0.389 | | -0.007 | 0.008 | [-0.020, 0.011] | 0.394 | |
| Age2 |  | -0.006 | 0.002 | [-0.010, -0.002] | 0.001 | | -0.048 | 0.015 | [-0.076, -0.018] | 0.001 | |
| Sex |  | -0.137 | 0.031 | [-0.199, -0.078] | < 0.001 | | -0.068 | 0.015 | [-0.099, -0.039] | < 0.001 | |
| Framewise Displacement |  | 1.027 | 0.149 | [0.734, 1.307] | < 0.001 | | 0.154 | 0.015 | [0.123, 0.182] | < 0.001 | |
| Scanner Type |  | 0.136 | 0.075 | [0.035, 0.344] | 0.071 | | 0.090 | 0.035 | [0.029, 0.167] | 0.009 | |
| Race |  | 0.018 | 0.012 | [-0.006, 0.040] | 0.133 | | 0.024 | 0.016 | [-0.008, 0.051] | 0.128 | |
| CON_DMN0 |  | 7.584 | 0.234 | [7.188, 8.139] | < 0.001 | | 0.406 | 0.013 | [0.382, 0.437] | < 0.001 | |
| ND |  | -0.061 | 0.015 | [-0.091, -0.033] | < 0.001 | | -0.061 | 0.015 | [-0.090, -0.032] | < 0.001 | |
| EU × Sex × ND |  | -0.025 | 0.19 | [-0.058, 0.015] | 0.190 | | -0.018 | 0.014 | [-0.044, 0.011] | 0.195 | |
| EU | CON_FPN2 | -0.006 | 0.010 | [-0.024, 0.016] | 0.582 | | -0.006 | 0.010 | [-0.023, 0.017] | 0.576 | |
| Threat |  | 0.009 | 0.007 | [-0.005, 0.024] | 0.224 | | 0.014 | 0.011 | [-0.008, 0.036] | 0.213 | |
| Age2 |  | -0.004 | 0.002 | [-0.007, -0.001] | 0.006 | | -0.035 | 0.013 | [-0.057, -0.009] | 0.006 | |
| Sex |  | -0.134 | 0.019 | [-0.171, -0.098] | < 0.001 | | -0.067 | 0.009 | [-0.085, -0.049] | < 0.001 | |
| Framewise Displacement |  | 0.359 | 0.108 | [0.159, 0.566] | 0.001 | | 0.054 | 0.014 | [0.025, 0.081] | < 0.001 | |
| Scanner Type |  | 0.089 | 0.028 | [0.027, 0.136] | 0.001 | | 0.059 | 0.019 | [0.021, 0.092] | 0.002 | |
| Race |  | 0.015 | 0.010 | [-0.004, 0.033] | 0.127 | | 0.019 | 0.012 | [-0.005, 0.043] | 0.118 | |
| CON_FPN0 |  | 9.144 | 0.339 | [8.447, 9.757] | < 0.001 | | 0.407 | 0.013 | [0.380, 0.430] | < 0.001 | |
| ND |  | -0.016 | 0.016 | [-0.048, 0.017] | 0.328 | | -0.016 | 0.016 | [-0.049, 0.016] | 0.330 | |
| EU × Sex × ND |  | -0.001 | 0.014 | [-0.027, 0.028] | 0.963 | | 0.000 | 0.010 | [-0.020, 0.020] | 0.963 | |
| EU | DMN_FPN2 | -0.007 | 0.014 | [-0.035, 0.020] | 0.635 | | -0.007 | 0.014 | [-0.033, 0.021] | 0.630 | |
| Threat |  | -0.003 | 0.007 | [-0.017, 0.011] | 0.643 | | -0.005 | 0.011 | [-0.028, 0.017] | 0.646 | |
| Age2 |  | 0.004 | 0.001 | [0.002, 0.006] | < 0.001 | | 0.030 | 0.008 | [0.014, 0.046] | < 0.001 | |
| Sex |  | 0.032 | 0.017 | [0.002, 0.069] | 0.053 | | 0.016 | 0.008 | [0.001, 0.034] | 0.053 | |
| Framewise Displacement |  | 0.609 | 0.084 | [0.458, 0.786] | < 0.001 | | 0.091 | 0.011 | [0.070, 0.111] | < 0.001 | |
| Scanner Type |  | 0.077 | 0.041 | [0.004, 0.162] | 0.062 | | 0.051 | 0.024 | [0.006, 0.097] | 0.032 | |
| Race |  | 0.027 | 0.012 | [0.007, 0.054] | 0.020 | | 0.036 | 0.015 | [0.010, 0.069] | 0.015 | |
| DMN_FPN0 |  | 10.589 | 0.385 | [9.922, 11.417] | < 0.001 | | 0.450 | 0.013 | [0.426, 0.476] | < 0.001 | |
| ND |  | -0.033 | 0.017 | [-0.073, -0.004] | 0.060 | | -0.033 | 0.017 | [-0.070, -0.005] | 0.052 | |
| EU × Sex × ND |  | 0.026 | 0.016 | [-0.003, 0.061] | 0.115 | | 0.019 | 0.013 | [-0.002, 0.047] | 0.136 | |

Note. EU = Environmental Unpredictability, ND = Overall Neighborhood Deprivation, CON = average correlations within the Cingulo-Opercular Network, DMN = average correlations within the Default Mode Network, FPN = average correlations within the Fronto-Parietal Network, CON_DMN = average correlations between Cingulo-Opercular Network and Default Mode Network, CON_FPN = average correlations between Cingulo-Opercular Network and Fronto-Parietal Network, DMN_FPN = average correlations between Default Mode Network and Fronto-Parietal Network. And the number following the abbreviation of the variable name represents the time of data collection, 0 = baseline, 2 = 2-year follow-up. Due to resampling in the cluster-robust standard error model, model fit indices (e.g., chi-square, CFI, and TLI) were not available with replicate weights. Based on the available indices (RMSEA = 0.000, SRMR = 0.000), the models fitted well. The reported *p* values were uncorrected.

Table S13. Mediating Effect of Changes in rsFC between Environmental Unpredictability and Children’s Internalizing/Externalizing Problems.

| Mediation Model | Model Fit | | Unstandardized | | | | Standardized | | | |
| --- | --- | --- | --- | --- | --- | --- | --- | --- | --- | --- |
|  | RMSEA [90%CI] | SRMR | Indirect Effect | *SE* | 95%CI | *p* | Indirect Effect | *SE* | 95%CI | *p* |
| EU→DMN→IP | 0.011 [0.000, 0.022] | 0.003 | 0.001 | 0.001 | [0.000, 0.002] | 0.427 | 0.000 | 0.001 | [0.000, 0.002] | 0.422 |
| EU→DMN→EP | 0.000 [0.000, 0.017] | 0.003 | 0.001 | 0.001 | [0.001, 0.002] | 0.041 | 0.001 | 0.000 | [0.000, 0.002] | 0.043 |
| EU→CON_DMN→IP | 0.006 [0.000, 0.019] | 0.003 | 0.001 | 0.001 | [0.001, 0.003] | 0.020 | 0.001 | 0.000 | [0.000, 0.002] | 0.020 |
| EU→CON_DMN→EP | 0.007 [0.000, 0.019] | 0.003 | 0.002 | 0.001 | [0.001, 0.004] | 0.007 | 0.001 | 0.000 | [0.001, 0.002] | 0.007 |

Note. EU = Environmental Unpredictability, DMN = average correlations within the Default Mode Network, CON_DMN = average correlations between Cingulo-Opercular Network and Default Mode Network, IP = Internalizing Problems, EP = Externalizing Problems. Due to resampling in the cluster-robust standard error model, model fit indices (e.g., chi-square, CFI, and TLI) were not available with replicate weights. Based on the available indices (RMSEA, SRMR), the models fitted well.

Table 14. Moderated Mediation Model of Neighborhood Educational Deprivation.

| Moderated Mediation Model | Model Fit | | Unstandardized | | | | Standardized | | | |
| --- | --- | --- | --- | --- | --- | --- | --- | --- | --- | --- |
|  | RMSEA [90%CI] | SRMR | Moderated Mediating Effect | *SE* | 95%CI | *p* | Moderated Mediating Effect | *SE* | 95%CI | *p* |
| EU×ND_E→DMN→IP | 0.015 [0.007, 0.024] | 0.004 | -0.001 | 0.001 | [-0.004, 0.001] | 0.476 | -0.001 | 0.001 | [-0.004, 0.001] | 0.453 |
| EU×ND_E→DMN→EP | 0.000 [0.000, 0.014] | 0.003 | -0.001 | 0.001 | [-0.004, 0.000] | 0.200 | -0.001 | 0.001 | [-0.004, 0.000] | 0.161 |
| EU×ND_E→FPN→IP | 0.016 [0.007, 0.025] | 0.005 | -0.001 | 0.001 | [-0.003, 0.000] | 0.067 | -0.001 | 0.001 | [-0.003, 0.000] | 0.055 |
| EU×ND_E→FPN→EP | 0.000 [0.000, 0.015] | 0.003 | -0.001 | 0.001 | [-0.003, 0.000] | 0.054 | -0.001 | 0.001 | [-0.003, 0.000] | 0.041 |
| EU×ND_E→CON_DMN→IP | 0.014 [0.003, 0.023] | 0.004 | -0.001 | 0.001 | [-0.004, -0.001] | 0.036 | -0.001 | 0.001 | [-0.003, -0.001] | 0.028 |
| EU×ND_E→CON_DMN→EP | 0.003 [0.000, 0.016] | 0.003 | -0.002 | 0.001 | [-0.004, -0.001] | 0.032 | -0.002 | 0.001 | [-0.004, -0.001] | 0.023 |

Note. EU = Environmental Unpredictability, ND_E = Neighborhood Deprivation in Education domain, DMN = average correlations within the Default Mode Network, FPN = average correlations within the Fronto-Parietal Network, CON_DMN = average correlations between Cingulo-Opercular Network and Default Mode Network, IP = Internalizing Problems, EP = Externalizing Problems. Due to resampling in the cluster-robust standard error model, model fit indices (e.g., chi-square, CFI, and TLI) were not available with replicate weights. Based on the available indices (RMSEA, SRMR), the models fitted well.

Table S15. Associations between Environmental Unpredictability and Changes in rsFC after Handling Outliers.

| Independent Variable and Covariates | Dependent Variable | Unstandardized | | | | Standardized | | | | |  |
| --- | --- | --- | --- | --- | --- | --- | --- | --- | --- | --- | --- |
|  |  | Coefficient | *SE* | *95% CI* | *p* | | Coefficient | *SE* | *95% CI* | *p* | |
| Environmental Unpredictability | CON2 | 0.000 | 0.000 | [-0.001, 0.000] | 0.106 | | -0.020 | 0.012 | [-0.045, 0.004] | 0.104 | |
| Threat |  | -0.001 | 0.001 | [-0.002, 0.000] | 0.082 | | -0.020 | 0.011 | [-0.041, 0.002] | 0.074 | |
| Scanner Type |  | -0.002 | 0.002 | [-0.007, 0.002] | 0.368 | | -0.021 | 0.020 | [-0.057, 0.018] | 0.281 | |
| Framewise Displacement |  | -0.053 | 0.009 | [-0.072, -0.035] | < 0.001 | | -0.097 | 0.015 | [-0.129, -0.068] | < 0.001 | |
| Age2 |  | 0.000 | 0.000 | [0.000, 0.000] | 0.020 | | 0.022 | 0.010 | [0.002, 0.041] | 0.020 | |
| Race |  | -0.002 | 0.001 | [-0.003, -0.001] | 0.007 | | -0.033 | 0.012 | [-0.057, -0.012] | 0.004 | |
| Sex |  | 0.005 | 0.002 | [0.001, 0.008] | 0.016 | | 0.035 | 0.014 | [0.004, 0.061] | 0.016 | |
| CON0 |  | 0.505 | 0.011 | [0.481, 0.525] | < 0.001 | | 0.509 | 0.013 | [0.481, 0.531] | < 0.001 | |
| Environmental Unpredictability | DMN2 | -0.001 | 0.000 | [-0.001, 0.000] | < 0.001 | | -0.042 | 0.010 | [-0.064, -0.024] | < 0.001 | |
| Threat |  | 0.000 | 0.000 | [-0.001, 0.000] | 0.323 | | -0.010 | 0.010 | [-0.031, 0.010] | 0.323 | |
| Scanner Type |  | -0.009 | 0.007 | [-0.025, -0.002] | 0.190 | | -0.096 | 0.047 | [-0.210, -0.029] | 0.040 | |
| Framewise Displacement |  | -0.044 | 0.008 | [-0.059, -0.030] | < 0.001 | | -0.096 | 0.014 | [-0.121, -0.066] | < 0.001 | |
| Age2 |  | 0.000 | 0.000 | [0.000, 0.000] | 0.230 | | 0.014 | 0.012 | [-0.007, 0.038] | 0.229 | |
| Race |  | -0.002 | 0.001 | [-0.003, -0.001] | 0.001 | | -0.040 | 0.012 | [-0.063, -0.018] | 0.001 | |
| Sex |  | 0.009 | 0.001 | [0.007, 0.011] | < 0.001 | | 0.076 | 0.009 | [0.059, 0.094] | < 0.001 | |
| DMN0 |  | 0.515 | 0.029 | [0.446, 0.557] | < 0.001 | | 0.497 | 0.028 | [0.429, 0.534] | < 0.001 | |
| Environmental Unpredictability | FPN2 | 0.000 | 0.000 | [0.000, 0.000] | 0.481 | | 0.006 | 0.009 | [-0.015, 0.022] | 0.480 | |
| Threat |  | -0.001 | 0.000 | [-0.001, 0.000] | 0.178 | | -0.015 | 0.011 | [-0.038, 0.006] | 0.180 | |
| Scanner Type |  | -0.002 | 0.003 | [-0.008, 0.002] | 0.520 | | -0.021 | 0.024 | [-0.069, 0.028] | 0.401 | |
| Framewise Displacement |  | -0.037 | 0.006 | [-0.050, -0.025] | < 0.001 | | -0.083 | 0.012 | [-0.106, -0.058] | < 0.001 | |
| Age2 |  | 0.000 | 0.000 | [0.000, 0.000] | 0.383 | | 0.010 | 0.011 | [-0.013, 0.031] | 0.384 | |
| Race |  | -0.001 | 0.001 | [-0.002, 0.000] | 0.243 | | -0.014 | 0.012 | [-0.039, 0.009] | 0.248 | |
| Sex |  | 0.007 | 0.001 | [0.004, 0.010] | < 0.001 | | 0.058 | 0.013 | [0.033, 0.082] | < 0.001 | |
| FPN0 |  | 0.532 | 0.015 | [0.502, 0.561] | < 0.001 | | 0.517 | 0.015 | [0.489, 0.546] | < 0.001 | |
| Environmental Unpredictability | CON_DMN2 | 0.001 | 0.000 | [0.000, 0.001] | < 0.001 | | 0.035 | 0.008 | [0.021, 0.050] | < 0.001 | |
| Threat |  | 0.000 | 0.000 | [-0.001, 0.001] | 0.757 | | -0.003 | 0.009 | [-0.020, 0.017] | 0.758 | |
| Scanner Type |  | 0.008 | 0.007 | [0.001, 0.025] | 0.279 | | 0.090 | 0.054 | [0.009, 0.213] | 0.096 | |
| Framewise Displacement |  | 0.080 | 0.010 | [0.062, 0.101] | < 0.001 | | 0.182 | 0.017 | [0.149, 0.215] | < 0.001 | |
| Age2 |  | 0.000 | 0.000 | [-0.001, 0.000] | < 0.001 | | -0.045 | 0.013 | [-0.072, -0.021] | 0.001 | |
| Race |  | 0.001 | 0.001 | [0.000, 0.003] | 0.037 | | 0.034 | 0.016 | [0.000, 0.062] | 0.032 | |
| Sex |  | -0.007 | 0.002 | [-0.010, -0.003] | < 0.001 | | -0.060 | 0.015 | [-0.089, -0.030] | < 0.001 | |
| CON_DMN0 |  | 0.425 | 0.016 | [0.393, 0.454] | < 0.001 | | 0.404 | 0.015 | [0.373, 0.431] | < 0.001 | |
| Environmental Unpredictability | CON_FPN2 | 0.000 | 0.000 | [0.000, 0.000] | 0.867 | | -0.002 | 0.011 | [-0.025, 0.018] | 0.866 | |
| Threat |  | 0.000 | 0.000 | [0.000, 0.001] | 0.265 | | 0.014 | 0.012 | [-0.011, 0.037] | 0.258 | |
| Scanner Type |  | 0.004 | 0.003 | [0.001, 0.011] | 0.214 | | 0.058 | 0.029 | [0.010, 0.118] | 0.044 | |
| Framewise Displacement |  | 0.026 | 0.006 | [0.015, 0.037] | < 0.001 | | 0.073 | 0.015 | [0.044, 0.101] | < 0.001 | |
| Age2 |  | 0.000 | 0.000 | [0.000, 0.000] | 0.010 | | -0.032 | 0.012 | [-0.056, -0.008] | 0.009 | |
| Race |  | 0.001 | 0.000 | [0.000, 0.002] | 0.122 | | 0.021 | 0.013 | [-0.005, 0.048] | 0.111 | |
| Sex |  | -0.006 | 0.001 | [-0.008, -0.004] | < 0.001 | | -0.064 | 0.010 | [-0.084, -0.044] | < 0.001 | |
| CON_FPN0 |  | 0.415 | 0.012 | [0.390, 0.436] | < 0.001 | | 0.405 | 0.013 | [0.378, 0.428] | < 0.001 | |
| Environmental Unpredictability | DMN_FPN2 | 0.000 | 0.000 | [0.000, 0.000] | 0.691 | | -0.006 | 0.015 | [-0.034, 0.025] | 0.688 | |
| Threat |  | 0.000 | 0.000 | [-0.001, 0.001] | 0.651 | | -0.005 | 0.012 | [-0.028, 0.017] | 0.653 | |
| Scanner Type |  | 0.003 | 0.003 | [0.000, 0.014] | 0.281 | | 0.051 | 0.031 | [-0.002, 0.113] | 0.100 | |
| Framewise Displacement |  | 0.036 | 0.005 | [0.026, 0.050] | < 0.001 | | 0.103 | 0.012 | [0.079, 0.127] | < 0.001 | |
| Age2 |  | 0.000 | 0.000 | [0.000, 0.000] | < 0.001 | | 0.032 | 0.009 | [0.015, 0.049] | < 0.001 | |
| Race |  | 0.001 | 0.001 | [0.000, 0.004] | 0.038 | | 0.043 | 0.019 | [0.008, 0.083] | 0.028 | |
| Sex |  | 0.002 | 0.001 | [0.000, 0.004] | 0.053 | | 0.017 | 0.009 | [0.001. 0.035] | 0.051 | |
| DMN_FPN0 |  | 0.471 | 0.014 | [0.443, 0.503] | < 0.001 | | 0.449 | 0.013 | [0.426, 0.476] | < 0.001 | |

Note. CON = average correlations within the Cingulo-Opercular Network, DMN = average correlations within the Default Mode Network, FPN = average correlations within the Fronto-Parietal Network, CON_DMN = average correlations between Cingulo-Opercular Network and Default Mode Network, CON_FPN = average correlations between Cingulo-Opercular Network and Fronto-Parietal Network, DMN_FPN = average correlations between Default Mode Network and Fronto-Parietal Network. And the number following the abbreviation of the variable name represents the time of data collection, 0 = baseline, 2 = 2-year follow-up. Due to resampling in the cluster-robust standard error model, model fit indices (e.g., chi-square, CFI, and TLI) were not available with replicate weights. Based on the available indices (RMSEA = 0.000, SRMR = 0.000), the models fitted well. The reported *p* values were uncorrected.

Table S16. Moderating Effect of Neighborhood Educational Deprivation on the Association between Environmental Unpredictability and rsFC after Handling Outliers.

| Independent Variable and Covariates | Dependent Variable | Unstandardized | | | | Standardized | | | | |  |
| --- | --- | --- | --- | --- | --- | --- | --- | --- | --- | --- | --- |
|  |  | Coefficient | *SE* | *95% CI* | *p* | | Coefficient | *SE* | *95% CI* | *p* | |
| EU | CON2 | -0.016 | 0.012 | [-0.039, 0.008] | 0.174 | | -0.016 | 0.012 | [-0.040, 0.008] | 0.175 | |
| Threat |  | -0.012 | 0.007 | [-0.027, 0.002] | 0.103 | | -0.019 | 0.011 | [-0.041, 0.003] | 0.094 | |
| Scanner Type |  | -0.032 | 0.036 | [-0.097, 0.031] | 0.361 | | -0.021 | 0.020 | [-0.057, 0.019] | 0.272 | |
| Framewise Displacement |  | -0.749 | 0.132 | [-1.027, -0.504] | < 0.001 | | -0.096 | 0.015 | [-0.128, -0.067] | < 0.001 | |
| Age2 |  | 0.003 | 0.001 | [0.000, 0.005] | 0.019 | | 0.023 | 0.010 | [0.003, 0.041] | 0.019 | |
| Race |  | -0.023 | 0.009 | [-0.042, -0.007] | 0.012 | | -0.030 | 0.011 | [-0.054, -0.009] | 0.009 | |
| Sex |  | 0.071 | 0.029 | [0.008, 0.124] | 0.015 | | 0.036 | 0.015 | [0.004, 0.062] | 0.016 | |
| CON0 |  | 7.229 | 0.156 | [6.901, 7.506] | < 0.001 | | 0.507 | 0.012 | [0.480, 0.527] | < 0.001 | |
| ND_E |  | 0.020 | 0.013 | [-0.005, 0.045] | 0.138 | | 0.020 | 0.013 | [-0.004, 0.044] | 0.128 | |
| EU × ND_E |  | 0.010 | 0.012 | [-0.014, 0.031] | 0.407 | | 0.010 | 0.012 | [-0.013, 0.031] | 0.405 | |
| EU | DMN2 | -0.033 | 0.011 | [-0.054, -0.012] | 0.002 | | -0.033 | 0.010 | [-0.053, -0.012] | 0.001 | |
| Threat |  | -0.006 | 0.006 | [-0.019, 0.007] | 0.378 | | -0.009 | 0.010 | [-0.029, 0.010] | 0.377 | |
| Scanner Type |  | -0.145 | 0.110 | [-0.404, -0.037] | 0.186 | | -0.096 | 0.046 | [-0.208, -0.032] | 0.036 | |
| Framewise Displacement |  | -0.728 | 0.128 | [-0.975, -0.480] | < 0.001 | | -0.094 | 0.014 | [-0.119, -0.063] | < 0.001 | |
| Age2 |  | 0.002 | 0.001 | [-0.001, 0.005] | 0.208 | | 0.015 | 0.012 | [-0.006, 0.039] | 0.207 | |
| Race |  | -0.026 | 0.007 | [-0.042, -0.013] | 0.001 | | -0.034 | 0.010 | [-0.055, -0.017] | < 0.001 | |
| Sex |  | 0.156 | 0.018 | [0.123, 0.190] | < 0.001 | | 0.078 | 0.009 | [0.061, 0.095] | < 0.001 | |
| DMN0 |  | 8.603 | 0.476 | [7.507, 9.295] | < 0.001 | | 0.495 | 0.027 | [0.430, 0.531] | < 0.001 | |
| ND_E |  | 0.036 | 0.017 | [0.005, 0.069] | 0.033 | | 0.036 | 0.016 | [0.005, 0.068] | 0.030 | |
| EU × ND_E |  | 0.033 | 0.012 | [0.011, 0.058] | 0.007 | | 0.033 | 0.011 | [0.011, 0.055] | 0.003 | |
| EU | FPN2 | 0.010 | 0.010 | [-0.012, 0.026] | 0.312 | | 0.010 | 0.010 | [-0.012, 0.026] | 0.313 | |
| Threat |  | -0.009 | 0.007 | [-0.024, 0.005] | 0.202 | | -0.015 | 0.012 | [-0.038, 0.007] | 0.205 | |
| Scanner Type |  | -0.031 | 0.050 | [-0.151, 0.038] | 0.539 | | -0.020 | 0.026 | [-0.072, 0.029] | 0.427 | |
| Framewise Displacement |  | -0.645 | 0.113 | [-0.858, -0.432] | < 0.001 | | -0.083 | 0.013 | [-0.106, -0.057] | < 0.001 | |
| Age2 |  | 0.001 | 0.001 | [-0.002, 0.004] | 0.390 | | 0.010 | 0.011 | [-0.014, 0.031] | 0.391 | |
| Race |  | -0.013 | 0.008 | [-0.028, 0.002] | 0.110 | | -0.017 | 0.011 | [-0.040, 0.002] | 0.119 | |
| Sex |  | 0.117 | 0.026 | [0.066, 0.168] | < 0.001 | | 0.059 | 0.013 | [0.033, 0.083] | < 0.001 | |
| FPN0 |  | 9.217 | 0.265 | [8.711, 9.706] | < 0.001 | | 0.517 | 0.015 | [0.490, 0.546] | < 0.001 | |
| ND_E |  | -0.013 | 0.018 | [-0.044, 0.029] | 0.471 | | -0.013 | 0.018 | [-0.044, 0.029] | 0.469 | |
| EU × ND_E |  | 0.027 | 0.009 | [0.011, 0.047] | 0.002 | | 0.028 | 0.008 | [0.011, 0.044] | 0.001 | |
| EU | CON_DMN2 | 0.027 | 0.009 | [0.008, 0.044] | 0.004 | | 0.027 | 0.009 | [0.008, 0.044] | 0.005 | |
| Threat |  | -0.003 | 0.006 | [-0.013, 0.010] | 0.655 | | -0.004 | 0.009 | [-0.021, 0.016] | 0.656 | |
| Scanner Type |  | 0.136 | 0.125 | [0.012, 0.432] | 0.278 | | 0.090 | 0.054 | [0.011, 0.212] | 0.094 | |
| Framewise Displacement |  | 1.402 | 0.176 | [1.078, 1.777] | < 0.001 | | 0.181 | 0.017 | [0.146, 0.213] | < 0.001 | |
| Age2 |  | -0.006 | 0.002 | [-0.009, -0.003] | < 0.001 | | -0.046 | 0.013 | [-0.072, -0.020] | 0.001 | |
| Race |  | 0.022 | 0.010 | [0.002, 0.042] | 0.203 | | 0.030 | 0.013 | [0.003, 0.054] | 0.018 | |
| Sex |  | -0.123 | 0.030 | [-0.179, -0.064] | < 0.001 | | -0.061 | 0.015 | [-0.090, -0.032] | < 0.001 | |
| CON_DMN0 |  | 7.515 | 0.270 | [6.955, 9.017] | < 0.001 | | 0.402 | 0.014 | [0.373, 0.429] | < 0.001 | |
| ND_E |  | -0.029 | 0.020 | [-0.069, 0.009] | 0.145 | | -0.029 | 0.020 | [-0.067, 0.009] | 0.140 | |
| EU × ND_E |  | -0.027 | 0.010 | [-0.049, -0.008] | 0.009 | | -0.027 | 0.010 | [-0.047, -0.009] | 0.005 | |
| EU | CON_FPN2 | -0.003 | 0.012 | [-0.027, 0.019] | 0.821 | | -0.003 | 0.012 | [-0.026, 0.019] | 0.820 | |
| Threat |  | 0.009 | 0.008 | [-0.007, 0.024] | 0.266 | | 0.014 | 0.012 | [-0.011, 0.037] | 0.259 | |
| Scanner Type |  | 0.088 | 0.073 | [0.011, 0.241] | 0.226 | | 0.058 | 0.030 | [0.009, 0.117] | 0.049 | |
| Framewise Displacement |  | 0.566 | 0.123 | [0.333, 0.806] | < 0.001 | | 0.073 | 0.015 | [0.043, 0.100] | < 0.001 | |
| Age2 |  | -0.004 | 0.002 | [-0.007, -0.001] | 0.010 | | -0.032 | 0.012 | [-0.056, -0.008] | 0.010 | |
| Race |  | 0.016 | 0.010 | [-0.002, 0.039] | 0.120 | | 0.022 | 0.013 | [-0.003, 0.050] | 0.108 | |
| Sex |  | -0.128 | 0.021 | [-0.170, -0.087] | < 0.001 | | -0.064 | 0.010 | [-0.084, -0.045] | < 0.001 | |
| CON_FPN0 |  | 9.115 | 0.258 | [8.552, 9.574] | < 0.001 | | 0.405 | 0.013 | [0.378, 0.427] | < 0.001 | |
| ND_E |  | 0.000 | 0.015 | [-0.030, 0.029] | 0.977 | | 0.000 | 0.015 | [-0.030, 0.029] | 0.977 | |
| EU × ND_E |  | -0.004 | 0.008 | [-0.020, 0.013] | 0.596 | | -0.004 | 0.008 | [-0.020, 0.012] | 0.595 | |
| EU | DMN_FPN2 | -0.008 | 0.015 | [-0.038, 0.020] | 0.565 | | -0.008 | 0.014 | [-0.037, 0.021] | 0.561 | |
| Threat |  | -0.004 | 0.008 | [-0.019, 0.012] | 0.644 | | -0.006 | 0.012 | [-0.030, 0.019] | 0.647 | |
| Scanner Type |  | 0.077 | 0.071 | [0.000, 0.227] | 0.275 | | 0.051 | 0.030 | [0.000, 0.111] | 0.092 | |
| Framewise Displacement |  | 0.791 | 0.118 | [0.579, 1.047] | < 0.001 | | 0.102 | 0.012 | [0.078, 0.126] | < 0.001 | |
| Age2 |  | 0.004 | 0.001 | [0.002, 0.006] | < 0.001 | | 0.032 | 0.009 | [0.014, 0.049] | < 0.001 | |
| Race |  | 0.029 | 0.015 | [0.004, 0.063] | 0.055 | | 0.039 | 0.019 | [0.005, 0.081] | 0.043 | |
| Sex |  | 0.033 | 0.017 | [0.001, 0.068] | 0.057 | | 0.017 | 0.009 | [0.000, 0.034] | 0.055 | |
| DMN_FPN0 |  | 10.572 | 0.309 | [9.915, 11.131] | < 0.001 | | 0.449 | 0.013 | [0.425, 0.475] | < 0.001 | |
| ND_E |  | -0.023 | 0.016 | [-0.061, 0.006] | 0.161 | | -0.023 | 0.016 | [-0.058, 0.006] | 0.150 | |
| EU × ND_E |  | 0.002 | 0.012 | [-0.021, 0.026] | 0.863 | | 0.002 | 0.012 | [-0.021, 0.026] | 0.862 | |

Note. EU = Environmental Unpredictability, ND_E = Neighborhood Deprivation in Education domain, CON = average correlations within the Cingulo-Opercular Network, DMN = average correlations within the Default Mode Network, FPN = average correlations within the Fronto-Parietal Network, CON_DMN = average correlations between Cingulo-Opercular Network and Default Mode Network, CON_FPN = average correlations between Cingulo-Opercular Network and Fronto-Parietal Network, DMN_FPN = average correlations between Default Mode Network and Fronto-Parietal Network. And the number following the abbreviation of the variable name represents the time of data collection, 0 = baseline, 2 = 2-year follow-up. Due to resampling in the cluster-robust standard error model, model fit indices (e.g., chi-square, CFI, and TLI) were not available with replicate weights. Based on the available indices (RMSEA = 0.000, SRMR = 0.000), the models fitted well. The reported *p* values were uncorrected.

Table S17. Mediating Effect of Changes in rsFC between Environmental Unpredictability and Children’s Internalizing/Externalizing Problems after Handling Outliers.

| Mediation Model | Model Fit | | Unstandardized | | | | Standardized | | | |
| --- | --- | --- | --- | --- | --- | --- | --- | --- | --- | --- |
|  | RMSEA [90%CI] | SRMR | Indirect Effect | *SE* | 95%CI | *p* | Indirect Effect | *SE* | 95%CI | *p* |
| EU→DMN→IP | 0.010 [0.000, 0.021] | 0.003 | 0.001 | 0.001 | [-0.001, 0.003] | 0.485 | 0.000 | 0.001 | [-0.001, 0.002] | 0.484 |
| EU→DMN→EP | 0.007 [0.000, 0.020] | 0.003 | 0.002 | 0.001 | [0.000, 0.003] | 0.026 | 0.001 | 0.000 | [0.000, 0.002] | 0.030 |
| EU→CON_DMN→IP | 0.005 [0.000, 0.018] | 0.003 | 0.001 | 0.001 | [0.001, 0.003] | 0.026 | 0.001 | 0.000 | [0.000, 0.002] | 0.027 |
| EU→CON_DMN→EP | 0.010 [0.000, 0.022] | 0.004 | 0.002 | 0.001 | [0.001, 0.004] | 0.007 | 0.001 | 0.000 | [0.001, 0.003] | 0.008 |

Note. EU = Environmental Unpredictability, DMN = average correlations within the Default Mode Network, CON_DMN = average correlations between Cingulo-Opercular Network and Default Mode Network, IP = Internalizing Problems, EP = Externalizing Problems. Due to resampling in the cluster-robust standard error model, model fit indices (e.g., chi-square, CFI, and TLI) were not available with replicate weights. Based on the available indices (RMSEA, SRMR), the models fitted well.

Table 18. Moderated Mediation Model of Neighborhood Educational Deprivation after Handling Outliers.

| Moderated Mediation Model | Model Fit | | Unstandardized | | | | Standardized | | | |
| --- | --- | --- | --- | --- | --- | --- | --- | --- | --- | --- |
|  | RMSEA [90%CI] | SRMR | Moderated Mediation Effect | *SE* | 95%CI | *p* | Moderated Mediation Effect | *SE* | 95%CI | *p* |
| EU×ND_E→DMN→IP | 0.015 [0.006, 0.024] | 0.004 | -0.001 | 0.001 | [-0.003, 0.001] | 0.532 | -0.001 | 0.001 | [-0.003, 0.001] | 0.515 |
| EU×ND_E→DMN→EP | 0.006 [0.000, 0.017] | 0.003 | -0.002 | 0.001 | [-0.004, 0.000] | 0.161 | -0.002 | 0.001 | [-0.004, 0.000] | 0.134 |
| EU×ND_E→FPN→IP | 0.015 [0.006, 0.024] | 0.004 | -0.001 | 0.001 | [-0.003, 0.000] | 0.050 | -0.001 | 0.001 | [-0.003, 0.000] | 0.041 |
| EU×ND_E→FPN→EP | 0.008 [0.000, 0.018] | 0.003 | -0.001 | 0.001 | [-0.003, 0.000] | 0.043 | -0.001 | 0.001 | [-0.003, 0.000] | 0.033 |
| EU×ND_E→CON_DMN→IP | 0.013 [0.003, 0.023] | 0.004 | -0.001 | 0.001 | [-0.003, 0.000] | 0.034 | -0.001 | 0.001 | [-0.003, 0.000] | 0.030 |
| EU×ND_E→CON_DMN→EP | 0.008 [0.000, 0.019] | 0.003 | -0.002 | 0.001 | [-0.005, -0.001] | 0.025 | -0.002 | 0.001 | [-0.004, -0.001] | 0.019 |

Note. EU = Environmental Unpredictability, ND_E = Neighborhood Deprivation in Education domain, DMN = average correlations within the Default Mode Network, FPN = average correlations within the Fronto-Parietal Network, CON_DMN = average correlations between Cingulo-Opercular Network and Default Mode Network, IP = Internalizing Problems, EP = Externalizing Problems. Due to resampling in the cluster-robust standard error model, model fit indices (e.g., chi-square, CFI, and TLI) were not available with replicate weights. Based on the available indices (RMSEA, SRMR), the models fitted well.

**References**

Belsky, J., Schlomer, G. L., & Ellis, B. J. (2012). Beyond cumulative risk: distinguishing harshness and unpredictability as determinants of parenting and early life history strategy. *Developmental Psychology*, *48*(3), 662–673. doi:10.1037/a0024454

Cochrane, R., & Robertson, A. (1973). The life events inventory: a measure of the relative severity of psycho-social stressors. *Journal of Psychosomatic Research*, *17*(2), 135–140. doi:10.1016/0022-3999(73)90014-7

Fan, C. C., Marshall, A., Smolker, H., Gonzalez, M. R., Tapert, S. F., Barch, D. M., … Herting, M. M. (2021). Adolescent Brain Cognitive Development (ABCD) study Linked External Data (LED): Protocol and practices for geocoding and assignment of environmental data. *Developmental Cognitive Neuroscience*, *52*, 101030. doi: 10.1016/j.dcn.2021.101030

Graham, J. W. (2003). Adding missing-data-relevant variables to FIML-based structural equation models. *Structural Equation Modeling*, *10*(1), 80-100. doi:10.1207/S15328007SEM1001_4

Mittal, C., Griskevicius, V., Simpson, J. A., Sung, S., & Young, E. S. (2015). Cognitive adaptations to stressful environments: When childhood adversity enhances adult executive function. *Journal of Personality and Social Psychology*, *109*(4), 604–621. doi:10.1037/pspi0000028

Noelke, C., McArdle, N., Baek, M., Huntington, N., Huber, R., Hardy, E., & Acevedo-Garcia, D. (2020). Child Opportunity Index 2.0 Technical Documentation. Retrieved from diversitydatakids.org/research library/research-brief/how-we-built-it
